# Infectious disease, shifting climates, and opportunistic predators: cumulative factors potentially impacting wild salmon declines

**DOI:** 10.1111/eva.12164

**Published:** 2014-05-27

**Authors:** Kristina M Miller, Amy Teffer, Strahan Tucker, Shaorong Li, Angela D Schulze, Marc Trudel, Francis Juanes, Amy Tabata, Karia H Kaukinen, Norma G Ginther, Tobi J Ming, Steven J Cooke, J Mark Hipfner, David A Patterson, Scott G Hinch

**Affiliations:** 1 Pacific Biological Station, Fisheries and Oceans CanadaNanaimo, BC, Canada; 2 Forest and Conservation Sciences, University of British ColumbiaVancouver, BC, Canada; 3 Biology Department, University of VictoriaVictoria, BC, Canada; 4 Fisheries and Oceans Canada, School of Resource and Environmental Management, Simon Fraser University, Science BranchBurnaby, BC, Canada; 5 Environment Canada, Wildlife Research DivisionDelta, BC, Canada; 6 Fish Ecology and Conservation Physiology Laboratory, Department of Biology, Carleton UniverisyOttawa, ON, Canada

**Keywords:** climate, coevolution, cumulative impacts, ecological impacts, infectious disease, microparasite, predation, wild salmon

## Abstract

Emerging diseases are impacting animals under high-density culture, yet few studies assess their importance to wild populations. Microparasites selected for enhanced virulence in culture settings should be less successful maintaining infectivity in wild populations, as once the host dies, there are limited opportunities to infect new individuals. Instead, moderately virulent microparasites persisting for long periods across multiple environments are of greatest concern. Evolved resistance to endemic microparasites may reduce susceptibilities, but as barriers to microparasite distributions are weakened, and environments become more stressful, unexposed populations may be impacted and pathogenicity enhanced. We provide an overview of the evolutionary and ecological impacts of infectious diseases in wild salmon and suggest ways in which modern technologies can elucidate the microparasites of greatest potential import. We present four case studies that resolve microparasite impacts on adult salmon migration success, impact of river warming on microparasite replication, and infection status on susceptibility to predation. Future health of wild salmon must be considered in a holistic context that includes the cumulative or synergistic impacts of multiple stressors. These approaches will identify populations at greatest risk, critically needed to manage and potentially ameliorate the shifts in current or future trajectories of wild populations.

## Introduction

Pacific Salmon are iconic fish that not only provide great economic, cultural and social benefit to humans (Lichatowich 1999) but are considered keystone species due in part to the tremendous nutrients they provide to both terrestrial and aquatic ecosystems as both live prey and decomposing carcasses (Cederholm et al. 1999). As anadromous fish, Pacific salmon hatch in freshwater lakes and streams, typically migrating to the ocean after 3–24 months where they may travel thousands of kilometers to reach feeding grounds before returning as mature adults for a single spawning event in the same natal rearing areas in which they were born (see Groot and Margolis 1991 for summary of the immense variation in life history within this general framework). Their high fidelity to natal streams and lakes has created strong genetic segregation among populations shaped by both demography and selection, especially for species that migrate long distances upstream to spawn (e.g., Sockeye [*Oncorhynchus nerka*] and Chinook [*O. tshawytscha*] Salmon; Beacham et al. 2006a,b).

Productivity (measured as adults produced per spawner) of southern US populations of Coho (*O. kisutch*) and Chinook Salmon has been declining for decades; almost half of the most southerly distributed populations of Coho Salmon have become extirpated, while many others are listed as threatened or endangered (Nehlsen et al. 1991; Brown et al. 1994). In southern British Columbia, populations of Coho Salmon began declining in the 1980s, followed by Chinook Salmon in the late 1980s and Sockeye Salmon in the early 1990s (Beamish et al. 1995; Peterman and Dorner 2012; Beamish et al. 2012). Alternately, during this same period, Pink (*O. gorbuscha*) and Chum (*O. keta*) Salmon, both species that have the shortest duration of freshwater residency, have been increasing in productivity (Irvine and Fukuwaka 2011).

In Canada, owing to the high-profile Fraser River salmon populations, the changes in fish population abundances have garnered much public and political attention. Coincident with the general patterns of declining productivity have been greater annual fluctuations in numbers of fish returning to the fishery (Sharma et al. 2013) which are often not accurately predicted by current management models (Haeseker et al. 2008; Hinch et al. 2012; Grant et al 2010). Predicting returns of Sockeye Salmon have been the most problematic, with preseason forecasts (defined as the mid-point of the distribution of probable returns) off by 10s of millions of fish in some years (Peterman and Dorner 2011; Grant and MacDonald 2012). In 2009, Fraser River Sockeye Salmon experienced the lowest returns in over 60 years, with only 14% of the predicted 10.5 million returns arriving to the river (Peterman and Dorner 2011). This event combined with recent declines spurred Canada’s Prime Minister to call for a public inquiry into the Decline of Sockeye Salmon in the Fraser River (‘Cohen Commission’, www.cohencommission.ca/en/). The following year was just as anomalous, with >28 million fish returning to spawn (S. Grant, unpublished data), nearly three times the median predicted by the run size forecast models, but still within the forecast range (Grant et al. 2010).

The Cohen Commission of Inquiry was tasked with assessing the scientific evidence to determine the cause of the declines in Fraser River Sockeye Salmon productivity as well as reviewing management practices and how scientific information is utilized to inform management decisions (Cohen 2012a). Although no single ‘silver bullet’ cause for the declines was identified, climate change impacting early ocean rearing conditions, infectious disease, predators, and aquaculture were considered perhaps most important of proposed factors, with a strong recognition that multiple cumulative stressors, some which may interact, were likely involved. In his final report (Cohen 2012b), Cohen suggested that the supporting science needs to move from basic understanding of adaptive responses to single stressors to predictive tools that can integrate the effects of multiple stressors.

While the situation for Coho, Chinook and Sockeye Salmon in BC appears dire for many populations, the fact that some populations are still performing moderately well suggest that both plastic and evolutionary mechanisms are contributing to responses to stressors associated with declines in abundance. In this special issue, we were asked to provide new insight into the evolutionary and ecological role of infectious disease in wild populations. Herein, we provide an extensive review of the conceptual background and current state of knowledge surrounding infectious disease impacts on wild salmon populations, and the potential interplay between two additional stressors, temperature, and predators, which may associate with salmon declines and influence or be influenced by infectious disease. We restrict most of our focus to microparasites (viruses, bacteria, myxozoans, and some fungi), as their instability and ability to exponentially replicate over very short periods of time enhances their potential for associating with population-level impacts (Bakke and Harris 1998). This assertion is backed by several reviews of wildlife disease outbreaks around the world, for which very few have been caused by macroparasites (Dobson and Foufopoulos 2001; Lafferty and Gerber 2002). We present evidence for phenotypic variation among populations that may result in different outcomes from each of these stressors and explore the evolutionary mechanistic responses that have been demonstrated to date. We note that there is a bias in our examples toward wild salmon in BC. We then present four case studies that each present novel approaches to address hypotheses on ecological and evolutionary consequences of single and cumulative stressors involving infectious agents. These studies take a population approach rather than a traditional veterinary focus on diagnosis and treatment, similar to that of Lyles and Dobson (1993) and the review by Lafferty and Gerber (2002). These case studies all incorporate a broad-based molecular microparasite monitoring approach capable of assessing the presence and load of dozens of microparasites at once and were performed as a ‘proof of concept’ for a new multidisciplinary research program on BC salmon health intended to support Pacific salmon management and conservation.

## Synoptic review

We conducted an extensive literature review to put this section together and have chosen to focus the text more on conceptual discussion rather than on specific details about each microparasite. Key references for the conditions under which each of dozens of microparasites have been shown to impact salmon can be found in Table 1 and studies showing genetic associations with and transcriptional host responses to specific microparasites can be found in Table S1. While the tables are used extensively to demonstrate conceptual ideas in the text, we ask readers to refer to the tables themselves for pertinent references on specific microbes, as many are not repeated in the text; references only cited in the tables are provided under supplemental references.

**Table 1:** Microparasites known or suspected to cause disease or economic impact in salmon throughout the world.

Microbe	Agent	Abbrev.	Disease	Disease in salmon	Present in BC	Risk to Sockeye	Hatchery	Carrier State Detection	Epidemic /high loss Associations	High-Risk Europe	Introduced to Chile	FW juveniles	FW adults	SW	Temperature responsive	Swim performance	Feeding Growth	Osmoreg.
A																		
Aeromonas hydrophila	Bacteria	Ahyd	Hemorrhagic septicemia	Seshadri et al. (2006)	X		X	Markwardt et al. (1989)							McCullough (1999); Croz ier et al. (2008)			
Aeromonas salmonicida	Bacteria	Asal	Furunculosis	Reith et al. (2008)	X	H	C	Markwardt et al. (1989); Austin and Austin (1993)	Emmerich and Weibel (1894)		1995	Evelyn et al. (1998)	Kent (2011)	Kent (2011)	McCullough (1999); Crozier et al. (2008)	Evelyn et al. (1998)		
Flavobacterium psychrophilum	Bacteria	CWD	Cold-water disease	Duchaud et al. (2007)	X	M	X	Nylund et al. (2011); Stephen et al. (2011)	Duchaud et al. (2007)	Duesund et al. (2010)		Stephen et al. (2011)	Kent (2011)		Stephen et al. (2011)			
Flavobacterium columnare	Bacteria		Columnaris		X	M		Austin and Austin (1993)	Pacha and Ordal (1963)					Duesund et al. (2010)	Holt et al. (1975)			
Salmon (Gill) chlamydia	Bacteria	Sch		Duesund et al. (2010)				Duesund et al. (2010) (?)	Duesund et al. (2010)	Duesund et al. (2010)				Duesund et al. (2010)				
Piscichlamydia salmonis	Bacteria	Pch	Contributing cause of Proliferative gill disease (PGD)	Duesund et al. (2010)				Duesund et al. (2010) (?)		Duesund et al. (2010)				Duesund et al. (2010)				
Piscirickettsia salmonis	Bacteria	Psal	Salmonid rickettisal septicemia	Larenas et al. (2003)	X	L	NP	Larenas et al. (2003)	Larenas et al. (2003)		1988			Kent 2011;				
Renibacterium salmoninarum	Bacteria	Rs (BKD)	Bacterial kidney disease	Wiens et al. (2008)	X	H	X	Wood and Yasutake (1956); Bullock and Herman (1988)	Skall et al. (2005)		1987	Mesa et al. (1999)	Elliott et al. (2013)	Elliott et al. (2013)			Pirhonen et al. (2000)	Price and Schreck (2003)
Rickettsia-Like Organism	Bacteria	RLO	Strawberry disease	X	X						1994		Lloyd et al. (2011) (trout)		Lloyd et al. (2011) (trout)			
Vibrio anguillarum	Bacteria	Vang	Vibriosis	Kent (2011)	X	H	C	Frans et al. (2011) (*n*)	Actis et al.. (1999)	Miyamoto and Eguc hi (1996)	2005			Kent (2011)	Egidius et al (1986)			Miyamoto and Eguchi (1996)
Vibrio salmonicida	Bacteria	Vsa	Cold-water vibriosis	Kent (2011)	X	H			0ivind et al. (1989)		2005			Oivind et al. (1989)	Egidius et al. (1986); 0ivind et al. (1989)			
Yersinia ruckeri	Bacteria	ERM	Enteric redmouth	Glenn et al. (2011)	X		X	Glenn et al. (2011)	Glenn et al. (2011)			Stephen et al. (2011)	Glenn et al. (2011)	Glenn et al. (2011)				
B																		
Atlantic salmon paramyxovirus	Virus	ASPV	Contributing cause of proliferative gill inflammation (PGI)	Kvellestad et al. (2005)					Kvellestad et al. (2005)	Kvellstad et al. (2005)				Kvellestad et al. (2005)	Kvellestad et al. (2005)		Kvellestad et al. (2005)	
Erythrocytic necrosis virus	Virus	ENV	Viral erythrocytic necrosis (VEN)	Evelyn and Traxler (1978)	X	L	X		Evelyn and Traxler (1978)					Kent (2011)			Evelyn and Traxler (1978)	Haney et al. (1992)
Infectious hematopoietic necrosis virus	Virus	IHNV	IHN	Wertheimer and Winton (1982)	X	H	X	St-Hilaire et al. (2001)	Rucker et al. (1953); Traxler et al. (1998)			Traxler et al. (1997)	Traxler et al. (1997)	Traxler et al. (1997)	Hetrick et al. (1979); LaPatra et al. (1989)	Meyers (2006)		
Infectious pancreatic necros is virus	Virus	IPNV	IPN	Wolf (1988); Rønneseth et al. (2007)	X	L	X	Johansen and Sommer (2001); Ronneseth et al. (2012)	Bahar et al. (2013)	Wolf (1988)	(1998)	W olf (1988); Rønneseth et al. (2007)		Kent (2011)	Dobos and Roberts (1983)	Meyers (2006)	Meyers (2006)	
Infectious salmon anemia virus	Virus	ISAV	ISA	Nylund et al. (1994)				Plarre et al. (2005); Nylund et al. (2011)	Nylund et al. (1994)	Plarre et al. (2005)	(2001)			Thorud and Djupvik (1998)	Falk et al. (1997)	Meyers (2006)		
Pacific salmon parvovirus	Virus	PSPvV		?	Kent (2011)	UK												
Piscine myocarditis virus	Virus	PMCV	Cardiomyopathy syndrome (CMS)	Haugland et al. (2011)	? Brocklebank and Raverty (2002)				Løvoll et al. (2010)	Ferguson et al. (1990)		W iik-Nielson et al. (2012)	Wiik-Nielson et al. (2012)	Wiik- Nielsen et al. (2012)		Haugland et al. (2011)		
Piscine reovirus	Virus	PRV	Heart and Skeletal Muscle Inflammatory Syndrome (HSMI)	Markussen et al. (2013)	Kibenge et al. (2013)			Palacios et al. (2010)	Løvoll et al. (2010)	Kongtorp et al. (2004); Finstad et al. (2012)	(2010)	Wiik-Nielson et al. (2011)	Wiik-Nielsen et al. (2011); Garseth et al. (2013)	Wiik-Nielson et al. (2012)		Kongtorpet al. (2004, 2008)		
Salmon alphavirus 1, 2, and 3	Virus	SAV 1/2/3	Pancreas Disease (PD) and Sleeping Disease (SD)	Graham et al. (2012)				Anderson et al. (2007); Nylund et al. (2011)	Snow et al. (2010)	Graham et al. (2012); Karlsen et al. (2013)		Nylund et al. (2003)		Karlsen et al. (2006)			McLoughlin and Doherty (1998)	
Viral encephalopathy and retinopathy virus	Virus	VER/VNN	Piscine nodavirus disease	Korsnes et al. (2005)					Plumb and Hanson (2011) - marine fish				(White Sturgeon)	Plumb and Hanson (2011)	Korsnes et al. (2005)			
Viral hemorrhagic septicemia virus	Virus	VHSV	VHS	Olesen et al. (1991)	X	L	X	Duesund et al. (2010)	Skall et al. (2005); Bowser et al. (2009)	Wolf (1988)		de Kinkelin et al. (1980)	Winton et al. (1991)	Skall et al. (2005)		Meyers (2006)	Baulaurier et al. (2012)	
C																		
Gyrodactylus salaris	Ectoparasitic worm	Gyro		Johnsen and Jensen (1991); Mo (1994)			X		Johnsen and Jensen (1991); Mo (1994)	Malmberg (1993)		Stephen et al. (2011)	Soleng et al. (1998)					
Ichthyophthirius multifiliis	Ciliate	IMR (Ich)		Bradford et al. (2010)	X	H			Bradford et al. (2010)				Bradford et al. (2010), Kocan et al. (2004)		Kocan et al. (2009)	Tierney and Farrell (2004)	Erickson (1965), Kocan et al. (2009)	
Nanophyetus salmincola	Fluke			Ferguson et al. (2012)										Jacobson et al. (2008)		Ferguson et al. (2012)	Ferguson et al. (2012)	Ferguson et al. (2012)
Neoparamoeba perurans	Amoeba	AGD	Amoebic gill diseas e	Kent (2011)	X	L	X	Nylund et al. (2011)			2006	Stephen et al. (2011)	X					
Spironucleus salmonicida Desmozoon	Flagellate			X	X													
Lepeophtherii (syn Paranucleospora theridion)	Microsporidium	NUC		Nylund et al. (2011)	X			Nylund et al. (2011)						Duesund et al. (2010)				
Facilispora margolisi	Microsporidium			?	Jones et al. (2012)									Jones et al. (2012)				
Loma salmonae	Microsporidium	Loma	Microsporidial Gill Disease of Salm on (MGDS)	Magor (1987)	X	L	**X**					Shaw et al. (2000)	Shaw et al. (2000)	Kent et al. (1995)				Kent et al. (1995)
Nucleospora salmonis	Microsporidium	Nsp	Chronic severe lymphobla- stosis	Kent (2011)	X	L	NP	Foltz et al. (2009)			1992	Kent (2011)	Kent (2011)	Kent (2011)				
Ceratomyxa shasta	Myxozoan	Cs	Ceratomyxosis	Stocking et al. (2006); Ray et al. (2010)	X	L	X	Foott et al. (2007)	Hallett et al. (2012)			Kent (2011)	Bartholomew (2010)	Kent (2011)	Stocking et al. (2006); Bartholomew (2009)		Meyers (2006)	
Kudoa thyrsites	Myxozoan	Kud		No	X		X			Moran et al. (1999)				Moran et al. (1999)				
Myxobolus arcticus	Myxozoan		Brain myxobolosis	?	X	L		Quinn et al. (1987)				Quinn et al. (1987)	Quinn et al. (1987)	Quinn et al. (1987)		Molesand Heifetz (1998)	Moles and Heifetz (1998)	
Myxobolus cerebralis	Myxozoan	Myx-18	Whirling disease	Kent (2011)		L		Cavender et al. (2004)							El-Matbouli et al. (1998), Baldwin et al. (2000)	Moles and Heifetz (1998)		
Myxobolus insidiosus	Myxozoan			X										Ferguson et al. (2012)		Ferguson et al. (2012)	Ferguson et al. (2012)	Ferguson et al. (2012)
Parvicapsula kabatai	Myxozoan			Jones et al. (2006)	X								Jones et al. (2006)					
Parvicapsula minibicornis	Myxozoan	Pm		Bradford et al. (2010)	X	H		Foott et al. (2007)	Bradford et al. (2010)			Kent (2011)	Bradford et al. (2010), Kocan et al. (2004)	Kent (2011)	Bradford et al. (2010)	Wagner et al. (2005)	Ferguson et al. (2012)	Bradford et al. (2010)
Parvicapsula pseudobranchicola	Myxozoan	Parvi2	Parvic- capsulosis	Nylund et al. (2005)				Nylund et al. (2011)		(emerging) Nylund et al. (2005)				Nylund et al. (2005)		Nylund et al.. (2005)		
Tetracapsuloides bryosalmonae	Myxozoan	PKD/PKX		Kent (2011)	X	M		Clifton-Hadley et al (1986)	Clifton-Hadley et al. (1984)	Clifton-Hadley et al. (1984)		Clifton-Hadley et al. (1984)		Clifton-Hadley et al. (1986)	Clifton-Hadley et al. (1986)	Clifton-Hadley et al. (1984)	Clifton-Hadley et al. (1984)	Kent et al. (1995)
Ichthyophonus hoferi	Protozoan			Rahimian (1998)	X	M	NP	Rahimian (1998)	Sindermann (1957); Sindermann and Chenoweth (1993); McVicar (1999); Johnsen and Jensen (1991); Mo (1994)	Rahimian and Thulin (1996)		Uno (1990)	Uno (1990)	Uno (1990)	Kocan et al. (2009)	Koc an et al. (2009)		Uno (1990); Stephen et al. (2011)
Sphaerothecum destruens	Protozoan			Kent (2011)	X	L	NP					Kent (2011)	Kent (2011)	Kent (2011)				

Table contains literature references for bacteria (A), viruses (B), and other microparasites (C), providing evidence of the environments and conditions upon which each microparasite is associated with disease and enhanced pathogenicity, and the sublethal impacts on the physiology and behavior of the host. Microparasites known to be in British Columbia are noted with an x, while emerging or exotic microparasites have literature referenced. Risk to Sockeye, as noted by Kent (2011), is represented as high (H), moderate (M), and low (L). Microparasite confirmation in fish from hatcheries (x), net pens (NP) or culture, are as per Kent (2011) and Stephen et al. (2011);. Carrier state detection is noted with references for asymptomatic (a), chronic/passive (c), nonpathogenic serotype (*n*) and carriers where the microparasite may have contributed to death, but on the cause of death (?). Dates associated with microparasite introduction to Chile are from Ibieta et al. 2011. Genus and species names and ‘et al.’ are not italisized for readability. References cited in the Table but not referred to in the text are presented in Reference S1.

### Challenges facing the assessment of infectious disease impacts in wild fish

Disease-causing microparasites are an inherent and natural component of ecosystems, greatly outnumbering free-living organisms (Windsor 1998), and likely infect every organism on the planet (Poulin 1996). As a consequence, microparasites are considered to be one of the major selective forces driving evolution (Maynard Smith 1976; Eizaguirre and Lenz 2010). Wildlife epidemics are of increasing concern, with all major ecosystems on earth affected (Harvell et al. 1999; Dobson and Foufopoulos 2001).

In wild populations, it is difficult to isolate and quantify the effects of any single factor, such as infectious disease or environmentally induced stress, because we rarely observe wild fish die; they simply disappear (La and Cooke 2011). Moreover, it is generally assumed that weakened fish are the first to fall prey to the numerous avian, mammalian, and piscine predators, although direct demonstrations of this hypothesis are rare. Stress is known to play a role in fish disease outbreaks (Wedemeyer 1970); stressors above which animals are able to maintain homeostasis have deleterious consequences for survival (Barton 2002). Many infectious agents (hereafter microparasites or microbes) are opportunistic and do not impact survival unless fish are also stressed by other factors impacting immune system function, such as poor water quality or toxicants, which exacerbate (Barton et al. 1985) or attenuate (Pickering and Pottinger 1987) the cortisol response to a second stressor (Barton 2002). For example, the ubiquitous oomycete *Saprolegnia* generally invades fish that have been stressed or otherwise have weakened immune systems (Bruno and Wood 1999). Other microparasites may be associated with chronic infections that can impact behavior, condition, and performance, which may render fish less capable of continued migration and/or more vulnerable to predation or starvation. Even small effects of infectious agents on physiological state or behavior can potentially be critical to the fitness of wild fish if they impact energy allocation or the timing of key life-history events (Bakke and Harris 1998). For example, impacts on growth can affect smolting (Marschall et al. 1998), early marine survival (Beamish and Mahnken 2001; Beamish et al. 2004), and predation rates (Hostetter 2009) in salmon. Finally, microparasites that cause acute disease may only do so in certain life-history stages or in specific habitats (e.g., fresh water or salt water). Infectious hematopoietic necrosis virus (IHNV), endemic to wild Sockeye Salmon populations (Rudakova et al. 2007), is a good example; it can cause significant losses of fry and smolts in freshwater but diminishes to nearly undetectable levels in saltwater, often increasing in load in adult fish returning to spawn in freshwater, but not causing measurable disease (Traxler et al. 1997). Interestingly, this same virus is associated with devastating losses of Atlantic Salmon (*Salmo salar*) in ocean net pens (St-Hilaire et al. 2002; Saksida 2006).

Most of what is known about disease impacts on salmon comes from fish in culture, where mortality is evident and measurable (Kurath and Winton 2011). Salmon enhancement hatcheries are abundant in the northeastern Pacific, accounting for 15.3% of the production of Coho and 18.6% of Chinook Salmon in Canadian commercial and Georgia Strait sport fisheries (Cross et al. 1991). In the Atlantic, 88% of Atlantic Salmon returning to US waters originated from hatcheries (Naish et al. 2008). From mortality events in these and other hatcheries around the world, there is a reasonable understanding of freshwater diseases important in a high-density hatchery-rearing environment. Aquaculture salmon have been reared in open ocean net-pens since the 1970s in Europe and the East Coast of Canada and the United States, and the 1990s on Canada’s West Coast and have been the primary source of information on infectious diseases impacting salmon in the ocean. However, as aquaculture is largely restricted to Atlantic Salmon, with only small numbers of farms culturing Chinook and Coho Salmon, information on ocean diseases impacting Sockeye, Chum and Pink Salmon is almost completely lacking (Kent 2011).

Fish health research generally follows events that start with observable mortality. Using a traditional veterinary diagnostic approach, abnormal feeding and swimming behavior and clinical signs of disease may be noted, followed by attempts at laboratory culture of infectious agents, histopathology to identify damage at the cellular level, and enzyme-linked immunosorbent assays and/or PCR of suspect microparasites. In the event that an infectious agent is suspected but not identified, degenerate PCR sequencing may be attempted if there are suspected microparasites. Challenge studies may also be pursued to demonstrate that the disease observed in association with mortality is, in fact, infectious. *In situ* hybridization can be used to identify whether suspected infectious agents are associated with regions of tissue damage. If an infectious agent is identified, challenge research will follow the guidelines set out by Koch’s postulates (1891) to establish a cause and effect relationship between the microparasite and clinical signs of disease. However, negative effects of subclinical infections in research are rarely reported (Kent et al. 2012).

Koch’s postulates were updated by Fredericks and Relman (1996) to incorporate modern molecular technologies as a powerful means for identifying yet to be cultured microparasites and for studying the host–parasite relationships. Previous to the advent of next-generation sequencing (NGS), microparasites that were difficult to culture could exist for decades with no identified agent. Two heart diseases, heart and skeletal muscle inflammatory syndrome (HSMI) and cardiomyopathy syndrome (CMS), impacted the European aquaculture industry for at least a decade before viral agents were discovered [piscine reovirus (PRV) - Palacios et al. 2010; piscine myocarditis virus (PMCV) - Haugland et al. 2011]. In the northeastern Pacific, erythrocytic necrosis (EN) has been associated with mortality in Chum and Pink Salmon for over three decades (Evelyn and Traxler 1978), and while inclusion bodies visible with histology could be used to determine the presence/absence of the disease (Arkoosh et al. 2004), the sequence of the virus causing the disease was obtained only this past year (ENV; J. Winton, USGS, personal communication).

Even with the revised postulates, establishing a direct cause and effect relationship between microparasites and disease may not be possible in wild populations if pathogenicity of an infectious agent causes infected fish to die and disappear before they are detected (Bakke and Harris 1998). Hence, despite abundant research on infectious disease impacts on fish in culture, our understanding of the ecological and evolutionary role of diseases impacting wild salmon populations is minimal (Bakke and Harris 1998; Kent 2011). Modeling studies assessing factors that may influence population fluctuations have implicated the potential role of disease (e.g., Levy and Wood 1992; Connors et al. 2012; Fujiwara et al. 2014), but empirical research to identify specific infectious diseases that could shift population trajectories is limited.

The complex life history of anadromous salmon may blur the effects of disease epidemics and make them harder to detect (Bakke and Harris 1998). As there is limited population-level monitoring for most salmon in BC, mortality that occurs during downstream river migration of smolts is often amalgamated with ocean mortality. Biotelemetry studies have recently shown that significant losses (up to 50%) can occur during downstream migration in two of the largest drainages in North America, the Fraser River in BC and the Columbia River in Washington/Oregon (Welch et al. 2009; Rechisky et al. 2013). Whereas in the Columbia River, downstream migration mortality is assessed regularly to address impacts of dams and alternate smolt transport systems (Schaller and Petrosky 2007), in BC, these opportunities are completely missed.

Over their highly migratory lifecycle, salmon may not only serve as vectors that can move microparasites from one environment to another (Walker and Winton 2010), but during the physiologically demanding shifts between freshwater, estuarine and marine ecosystems (Clarke and Hirano 1995), migrating salmon are also exposed to a suite of new microparasites carried in diverse host reservoirs, some of which may subsequently impact their performance. Importantly, it is during these transition periods when some studies speculate that mortalities can reach very high levels in a short period of time (Bradford 1995; Beamish et al. 2010), potentially high enough to exert strong evolutionary pressure on a population. Moreover, during these transition periods salmon from disparate environments converge, densities are maximized, and hormonal changes can cause immunosuppression (smolts - Maule et al. 1987; adults - Pickering and Christie 1980, 1981), providing an ideal environment for enzootic outbreaks of disease (Uno 1990). In southern BC populations of Sockeye, Chinook and Coho Salmon, levels of mortality in the early marine environment can be major determinants of year-class strength (Beamish and Mahnken 2001; LaCroix et al. 2009). It is during this critical early marine period that many believe the key to declining productivity lies (Beamish et al. 2010; Peterman and Dorner 2011). While climate-driven ocean conditions are hypothesized to play a major role (Chittenden et al. 2009; Rogers and Schindler 2011; Sharma et al. 2013), if disease were to contribute substantially to these mortalities in some or all years, genetic variance in susceptibilities to important disease-causing microparasites may underlie some of the population-level variances in returns. Importantly, density dependence is also strongly correlated with ocean productivity shifts (Elliott 1989), consistent with patterns expected if disease were a factor. However, as dying fish are virtually never observed, direct linkages with disease can be difficult to demonstrate. At the other end of the salmon life cycle, adult Pacific salmon migrate from the marine environment back to the freshwater rivers to spawn in the streams and tributaries in which they were born. As semelparous species, returning Pacific salmon are simultaneously maturing, senescing, and starving, and hence, their condition and ability to fight infection is deteriorating over the last stretches of their migration, making them especially vulnerable to additional environmental stressors and disease. Immunosupression induced by maturation hormones (Pickering and Christie 1980) may also contribute to enhanced susceptibility by even opportunistic microparasites or those previously at a carrier state. In recent decades, the level of premature mortality experienced by salmon in major drainages in BC and Washington has escalated coincident with the general 2–3°C rise in river temperatures (Patterson et al. 2007; Keefer et al. 2008; Martins et al. 2011). For example, premature mortality for Sockeye Salmon returning to the Fraser River to spawn was historically close to 15–20% but has been upward of 95% in some years, often showing a high degree of genetic variation among populations within the drainage (Hinch et al. 2012). It is somewhat easier to associate these mortality events with infectious diseases, as some of the mortalities are observable as carcasses full of eggs lining the riverbanks. However, complex infections with multiple microparasites can obscure assigning a single disease as a cause of death; case studies I–III, presented below, delve into the complexity of microparasites carried by salmon returning to spawn.

### Infectious disease impacts in wild salmon—what is known

Population-level effects of infectious disease have been observed in wild freshwater and marine fishes, but not commonly in salmon (Kent 2011) possibly due to the reasons stated previously. Classic cases of disease epidemics in fish include widespread outbreaks of viral hemorrhagic septicemia (VHS) in several fish species in the Great Lakes (Bowser et al. 2009) and herring (*Clupea pallasi*), hake (*Merluccius productus*), and walleye pollock (*Theragra chalcogramma*) in the northeastern Pacific (Skall et al. 2005), a herpes virus introduced to Australian pilchards (*Sardinops sagax*) in the 1990s by bait fish (Murray et al. 2003) and causing mass mortalities over thousands of kilometers (Jones et al. 1997), sturgeon (*Acipenser nudiventris*) population crashes in the Aral Sea after introduction of *Nitzschia sturionis* (Bauer 1961) and chronic *Ichthyophonus hoferi* infections causing high mortalities in herring worldwide (Sindermann and Chenoweth 1993; Rahimian and Thulin 1996). The first record of epidemic disease in wild salmon was from a paper dating to the late nineteenth century documenting furunculosis outbreaks (caused by bacterium *Aeromonas salmonicida*) in Atlantic Salmon (Emmerich and Weibel 1894). Subsequently, outbreaks of furunculosis (Inglis et al. 1993), ulcerative dermal necrosis (UDN; Roberts 1993), and *Gyrodactylus salaris* (Johnsen and Jensen 1991; Mo 1994) have caused widespread conspicuous epidemics in wild populations of Atlantic Salmon in Europe. As well, the bacterium *Renibacterium salmoninarum* caused a major epidemic of bacterial kidney disease in Scotland in the 1930’s (Smith 1964). In Pacific, salmon *Ichthyophonus* (Traxler et al. 1998) is suspected of associating with population-level impacts in the marine environment, while in freshwater, population-level mortality events have also been associated with *Ceratomyxa shasta* (Hallet et al. 2012), *Parvicapsula minibicornis* (Bradford et al. 2010) and *Ichthyophthirius multifiliis* (Kocan et al. 2004). Pacha and Ordal (1963) identified high *Flexibacter columnaris* infection rates as a potential cause for the decline of Columbia River Chinook, Sockeye, and Steelhead Trout (*Oncorhynchus mykiss*) in the early 1960s.

While macroparasites (defined as fish lice, tapeworms, nematodes, and some protozoan and fungal pathogens) can cause conspicuous harm to heavily infected individuals, they generally remain relatively stable over time and have limited impacts at the population level (sea lice may be an exception; Johnson et al. 1996; Krkošek et al. 2006) (Bakke and Harris 1998). Moreover, the complex life cycles of many macroparasites that require intermediate hosts to complete development further limits the range of environments where they can persist (Dobson and Foufopoulos 2001). Alternately, microparasites (e.g., viruses, bacteria, some protozoan, and some fungi) are very unstable, exponentially increasing over very short periods of time, and have a much greater potential as regulators of host population size and as selective agents (Bakke and Harris 1998). Given their volatile nature, microparasites are also associated with stronger immune responses that result in lasting immunity (Anderson and May 1979). For wild Norwegian Atlantic Salmon, a review by Bakke and Harris (1998) concluded that myxozoans, furunculosis, *G. salaris*, and sea lice are the pathogens of greatest threat. While viral diseases are common in cultured European salmon, they argued that there was no evidence of viral disease impacts on wild salmon, or of transfer of viruses from farmed to wild fish. In Pacific salmon off North America, a similar assessment of risk for population-level impacts of disease in Sockeye Salmon was conducted by Kent (2011). Microparasites identified as ‘high risk’ included the IHN virus, well known to cause significant disease in juvenile Sockeye Salmon (Traxler et al. 1997), bacterial species *A. salmonicida* and *R. salmoninarum* that have been associated with highly observable hatchery losses of Coho and Chinook Salmon (Evelyn et al. 1998), *Vibrio* (*Listonella*) *anguillarum*, a bacterium associated with high losses of Pacific salmon in net pens (Actis et al. 1999), and two microparasites, *P. minibicornis* and *I. multifiliis* that have been associated with pre-mature mortality of returning adult salmon (Kocan et al. 2004; Bradford et al. 2010; Table 1). Importantly, most microparasites that had never been assessed in Sockeye Salmon (of which there were many) were classified as ‘low risk’, and the review only included known endemics. Kent (2011) suggested that there was no evidence of exotic or uncharacterized salmon pathogens in BC. He also argued that because salmon would have evolved natural resistance to endemic microparasites, any associations of endemic microbes with declines would require enhanced susceptibility due to additional environmental stressors.

Some microparasites can transcend freshwater, estuarine and saltwater ecosystems, while others cannot (see Table 1 for full list and references). For some, pathogenicity may be diminished by the osmoregulatory demands associated with shifts between salinity environments, limiting their impacts to a single ecosystem. In other cases, like that for IHNV described previously, genetic variance in susceptibility of the host appears to drive patterns of differential virulence between ecosystems. Alternately, there are numerous microparasites that can be transmitted in one environment but become more virulent in another. Some of the most devastating emerging viruses in European salmon can be transmitted in freshwater ecosystems with no apparent ill effects on juveniles, but become virulent pathogens after entering the ocean [e.g., PRV (Løvoll et al. 2012) and PMCV (Wiik-Nielsen et al. 2012)]. Infectious salmon anemia virus (ISAV) is an exception, as it is hypothesized that the avirulent wild-type strain of the virus, HPR0, may be transmitted in freshwater but can readily mutate under conditions that are not well understood to become a virulent pathogen in the marine environment (Plarre et al. 2012). A third pattern of differential virulence among ecosystems is microparasites that are merely carried in the marine environment but become pathogenic during the energetically and physiologically challenging return migration of adult salmon to spawning grounds.

Sublethal effects of microparasites may be more detrimental to wild than cultured populations, as they may impact the ability to compete effectively for resources, to migrate to optimal environments for feeding and overwintering, and to put enough energy into maturation to succeed in their once in a lifetime opportunity to spawn. Behavioral shifts are often the first line of defense when animals are stressed and are designed to lessen the probability of death or metabolic costs incurred by maintaining physiological homeostasis (Olla et al. 1980). Swimming performance is the behavioral trait perhaps most universally affected when animals are stressed and condition of fish is compromised (Webb and Brett 1973; Wedemeyer et al. 1990), with impairments in performance a good predictor of survival (Thomas et al. 1964). Given recent findings that show enhanced robustness and disease resistance in fit fish (those that have undergone aerobic training exercises), one might surmise that the relatively fitter wild fish would have an advantage over sedentary cultured fish (Castro et al. 2011). However, when swim performance is compromised, the impacts on survival of wild fish will be greater. Effects on swim performance have been associated with a wide array of parasitic and viral infectious agents in salmon (see Table 1). Appropriate food resources may improve favorable disease outcomes, such as reduced impacts of HSMI where functional feeds (high lipid/DHL content) reduced the viral load and lessened the pathology in heart tissues (Martinez-Rubio et al. 2012). However, microbes that impact swim performance may also decrease feeding and growth in wild fish (Table 1). While impacts will be felt at most stages of development, there is mounting evidence that impacts of reduced feeding and growth on survival of wild salmon in the early marine environment may be quite substantial (Beamish and Mahnken 2001; Beamish et al. 2004; Farley et al. 2007).

Infectious agents that cause disease in gill and/or kidney tissue are often associated with impaired osmoregulation and may indirectly impact salmon survival during salinity transitions. Osmotic stress during saltwater acclimation is metabolically challenging and can affect multiple energy-intensive behavioral traits, including schooling, foraging activity, predator avoidance, and swimming performance, potentially increasing risk of predation (Järvi 1989; Handeland et al. 1996; Dieperink et al. 2002). Prolonged osmotic stress may reduce growth and increase susceptibility to opportunistic pathogens and additional stressors or at the extreme, result in complete osmotic failure and death. Osmoregulatory indices have been associated with reduced survival of adult salmon returning to spawn (Cooke et al. 2006; Crossin et al. 2009; Donaldson et al. 2010; Miller et al. 2011), and disease is one of the suspected drivers of this variation (Miller et al. 2011; Jeffries et al. 2012). Numerous microparasites have been associated with impaired osmoregulation, while others increase pathogenicity during smoltification (Table 1).

### Evolutionary drivers of disease resistance in salmon

It is expected that genetic diversity within host populations, especially associated with immune system processes, can buffer them against widespread epidemics (Altizer et al. 2003). Organisms with low disease response capability should be rapidly wiped from a population (Kronenberg et al. 1994), and hence, in the face of novel microparasite exposures, if populations are to remain viable they need to evolve resistance quickly. The cycle of adaptation and counter-adaptation between microparasites and hosts creates an oscillatory dynamic of host and parasite genotypic frequencies and has been depicted as an ‘evolutionary arms race’ described under the ‘Red Queen Hypothesis’ (Van Valen 1973; Altizer et al. 2003).

Antagonistic coevolution between endemic microparasites and their host populations has created a geographic mosaic in patterns of susceptibility of salmon to infectious diseases and is a potential driving force maintaining genetic variation in immune system processes (Bakke et al. 1990; Gjedrem et al. 1991). Salmon populations with historical exposure to particular diseases generally carry greater resistance to those diseases (Zinn et al. 1977; Bower et al. 1995; Bartholomew 1998; Miller and Vincent 2008). Moreover, populations that have coevolved with specific infectious microparasites may show lower heritabilities than newly exposed populations, limiting the pace of future adaptation (Crozier et al. 2008). Genetic associations with resistance measured as survival under challenge testing have been demonstrated for a wide range of salmon microparasites (reviewed in Ødegård et al. 2011) of viral, bacterial, and parasitic origin (references in Table S1). Heritabilities range between 0.14 (sea louse) to 0.62 (furunculosis) and are generally higher than those observed in livestock (Ødegård et al. 2011). Several studies have explored the genetic correlations between resistance against a variety of diseases; while most are positively correlated (Gjøen et al. 1997; Henryon et al. 2005), indicative of common immune-related resistance genes, others may be negatively correlated or show no correlation at all (Ødegård et al. 2007; Kjøglum et al. 2008).

#### Disease resistance and the major histocompatibility complex

The complexity and polymorphism of the immune system suggests that it is indispensable for survival and argues for the importance of infectious agents as a selective force in natural populations (Bakke and Harris 1998). As such, we expect that host species exposed to a variety of microparasites should harbor a diverse array of resistance alleles or a range of inducible defences (Altizer et al. 2003). However, while most association studies in salmon have calculated heritabilities via familial associations with resistance, few have identified the underlying genetic mechanisms conferring resistance. There have been a fair number of targeted studies assessing associations between disease resistance and major histocompatibility complex (MHC) genes. MHC molecules play a crucial role in T-cell-mediated adaptive immune responses by binding self and parasite-derived peptides for presentation to T-cells (Potts and Wakeland 1990; Hedrick 1994). MHC class I molecules bind peptides produced within cells (e.g., derived from viruses, some microparasites) and generally elicit a cytotoxic response, while class II molecules bind peptides of exogenous infectious agents (e.g. most bacteria and macroparasites) generally resulting in a humoral (antibody) response.

Given the critical role in immune recognition of infectious agents and unprecedented levels of diversity displayed by MHC molecules, the evolutionary dynamics of the MHC has become a paradigm for adaptively important genetic diversity that is of relevance in ecology, population biology, and conservation (Sommer 2005; Piertney and Oliver 2006). Pathogen-driven balancing selection—derived through overdominance, negative frequency dependence or temporal/spatial heterogeneity in pathogen pressure—is hypothesized to be the dominant force driving MHC evolution (Klein and O’huigin 1994; Parham and Ohta 1996; Hedrick and Kim 2000). It is expected that the maintenance of MHC diversity in wild populations assures resistance to a diverse array of microparasites, hence enhanced population viability (reviewed in Bernatchez and Landry 2003; Sommer 2005; Piertney and Oliver 2006; but see Radwan et al. 2010). We expect that in natural communities, adaptation to newly encountered microparasites or changes in microparasite virulence occurs on ecological rather than evolutionary timescales, necessitating selection based on pre-existing genetic variation, referred to as ‘standing genetic variation’ (Barrett and Schluter 2008). MHC alleles associated with resistance or susceptibility to specific infectious agents of salmon have been identified in numerous laboratory challenge studies (ISAV – Grimholt et al. 2003 and IHNV – Palti et al. 2001; Miller et al. 2004; *A. salmonicida* – Langefors et al. 2001; Lohm et al. 2002; *Piscirickettsia salmonis* – Gomez et al. 2011) most consistent with the action of directional selection imposed by a single pathogen. Only a single study by Arkush et al. (2002), in which a series of bacterial (*V. anguillarum*), viral (IHNV), and parasite (*Myxobolus cerebralis*) challenges were conducted on inbred and outbred Chinook Salmon, demonstrated stronger single-pathogen selection for heterozygosity than for a specific resistance allele (IHNV only). Hence, if pathogen-driven selection is the dominant mechanism maintaining diversity of MHC molecules, the action of multiple pathogens is likely required.

The role of MHC genes in the evolution of local adaptation of anadromous salmon to differing microparasite communities among natal streams and lakes is supported by their higher level of population divergence than derived from demographics alone (Miller et al. 2001; Eizaguirre and Lenz 2010; McClelland et al. 2013). MHC allelic distribution patterns within salmon populations vary considerably, with some populations showing distributions more even than expected under neutrality (evidence of balancing selection), some less even (evidence of directional selection), and others showing no deviations from neutral expectations (Landry and Bernatchez 2001; Miller et al. 2001; Aquilar and Garza 2006; Campos et al. 2006; Dionne et al. 2007; Consuegra et al. 2011; McClelland et al. 2013). In Sockeye Salmon, the dominant class I (UBA) and II (DAB) loci show fluctuating patterns of allelic distribution across the species range that are not correlated between loci, suggesting that different selective forces are at play (McClelland et al. 2013). Most populations showing evidence of directional selection contain a single-dominant allele that may be a resistance allele to a virulent infectious agent (McClelland et al. 2013). Over the entire range of Sockeye Salmon, there are only two alleles at the DAB locus observed at frequencies >90%, and one allele for UBA, and these are distributed across demographically distant populations (McClelland et al. 2013). Whether the same selective agents are responsible for maintaining each of these dominant alleles across distant populations is worth investigating in the future.

While numerous salmon population studies have contrasted allele frequency data for MHC and selectively neutral loci to demonstrate natural selection acting on the MHC over an ecological time scale (Miller and Withler 1997; Landry and Bernatchez 2001; Miller et al. 2001; Aquilar and Garza 2006; Dionne et al. 2007; Peters and Turner 2008; McClelland et al. 2013), few have demonstrated in natural systems direct associations with pathogen resistance. A series of field studies based on wild Canadian Atlantic Salmon populations in Quebec offer some of the first direct correlations between microbes and shifting MHC allele frequencies in a single generation in salmon. Dionne et al. 2007 identified an association between bacterial community diversity and MHC class II*β* diversity along a latitudinal thermal cline, similar to patterns originally observed in humans (Prugnolle et al. 2005). A subsequent study identified an association between a dominant myxozoan parasite and two MHC class II*β* alleles, one statistically associated with susceptibility to infection, and the other with resistance (Dionne et al. 2009). Over time, the frequency of the susceptibility allele and infection with the myxozoan parasite decreased, consistent with rapid pathogen-driven directional selection based on standing genetic variation. A similar study on juvenile European Atlantic Salmon documented shifts in MHC allele frequencies over a six-month period in the river, possibly indicative of pathogen-driven selection, although in this case, pathogens were not monitored (de Eyto et al. 2011).

#### Genome scans for QTL’s associated with disease resistance

Genomic scans for genetic loci quantitatively associated with disease resistance (dQTL) have recently been conducted for a small number of salmon diseases (see below; Table S1). Unlike the MHC association studies, a dQTL approach is not targeted, but rather assesses associations across hundreds to thousands of single nucleotide polymorphisms [SNPs] or microsatellite loci mapped evenly across the genome. This approach can be used to identify the genetic architecture of disease resistance for a given disease, including the number of significantly associated loci across the genome, their level of contribution, and whether epistatic relationships exist between loci (Kover and Caicedo 2001). Synthesis of dQTL’s across a range of diseases will reveal the species-level genetic architecture of disease resistance, identifying clusters of dQTL’s impacting resistance to multiple diseases. This approach has been used effectively to identify breeding schemes for agricultural species of interest (e.g., maize – Wisser et al. 2006).

The largest focus of dQTL research in salmon has been on two important viral diseases significantly impacting global aquaculture of Atlantic Salmon, ISA, and infectious pancreatic necrosis (IPN). QTL discovery and validation studies have been undertaken for each (Table S1). These studies identified single major QTL’s associated with resistance to each viral disease. For IPN, virtually all of the variation in resistance in both freshwater and seawater was associated with a single dQTL on linkage group 21 (Houston et al. 2010). For ISA, a powerful dQTL was identified in linkage group 8 (Moen et al. 2004, 2007). Lack of a fully curated salmon genome sequence hampers the precise identification of genes associated with resistance using a QTL approach (Davidson et al. 2010; NCBI ASM23337v1). However, a comparative genomics approach identified a candidate gene linked by synteny in tetraodon and medaka genomes to the major QTL for ISA resistance that codes for a major regulatory protein of several genes that have been implicated in the response to ISAV infection (Li et al. 2011). A dQTL study on VHS also identified a single dominant QTL conferring resistance in Rainbow Trout (Verrier et al. 2013a). A subsequent study found no genetic correlation of this QTL with resistance to another fish rhabdovirus, IHNV (Verrier et al. 2013b).

### Phenotypic variation in disease response through gene expression profiling

Damage is a central feature of infectious disease; the degree of damage caused to host tissue will impact the level of host response and the pathological outcome of disease (Casadevall and Pirofski 1999). As such, microparasites can be ranked based on the likelihood that they cause damage, and hence disease, as a function of the magnitude of the host response (Casadevall and Pirofski 1999). Gene expression profiling can elucidate the molecular basis of variation in susceptibility and response to disease derived from both plastic and genetic mechanisms. CDNA microarrays and Agilent oligonucleotide arrays offer a high throughput method to assess the activity of thousands to 10s of thousands of genes at once and are the mainstay of functional genomics research. Numerous salmon arrays have been developed in the past decade, the most recent of which are Agilent oligonucleotide arrays with 44 000 gene features spotted onto four subarrays on each slide (Taggart et al. 2008; Jantzen et al. 2011). Array technology has been applied to assess salmon host response to a large number of infectious agents, including virtually all of the ‘high impact’ and emerging viral diseases (e.g. IHN, ISA, CMS, HSMI, and pancrease disease [PD]), a few of the important bacterial diseases (furunculosis, vibriosis, and rickettsia), but very few parasitic diseases (except amoebic gill disease, whirling disease, PKD, and sea louse) (Table 1-disease names; Table S1-references).

Most disease-focussed microarray studies have identified genes and biological processes up- and down-regulated in response to a pathogen. More importantly, a small number of studies have contrasted responses in high and low susceptibility fish or pathogen strains of high and low virulence that can begin to unravel the mechanistic basis of resistance (Miller et al. 2007; Wynne et al. 2008; Purcell et al. 2009). Across virtually all viral challenge studies, a powerful and systemic induction of antiviral and interferon (IFN)-dependent genes has been correlated with viral load and degree of tissue damage (see Table S1), mirroring the important role of IFNs in orchestration of antiviral responses in mammals. However, the salmon IFN response was also stimulated in response to bacterium *P. salmonis* (Tacchi et al. 2011) and myxozoan *M. cerebralis* (Baerwald et al. 2008). As a general rule, resistant and susceptible hosts are responding using highly congruent profiles of genes, but the level of response increases with susceptibility and virulence. Hence, it appears that in many cases, increasing the power of the host response is not sufficient to resist disease. Instead, more subtle variations in the pathways stimulated may underlie the levels of susceptibility of the host. For IPNV, survivors generally elicited a stronger innate immune responses (Marjara et al. 2011), whereas for IHNV, the efficiency of viral entry and strength of host down-regulation of cell transcription and translation appeared to be more important determinates of susceptibility (Miller et al. 2007 and K. M. Miller, unpublished data; Purcell et al. 2011). Alternately, the strength of complement activation appeared to be more predictive of resistance to bacteria *A*. *salmonicida* (Škugor et al. 2009) and *Flavobacterium psychrophilum* (Langevin et al. 2012). A single study on ISAV contradicted the pattern of enhanced response with higher microbe loads and more susceptible hosts; Workenhe et al. (2009) found that a low virulent strain of ISAV elicited a stronger host response than highly virulent strains.

In the second case study described below, we combine quantitative data on microparasites carried by wild migrating salmon with a measure of host response defined by the transcriptional activity of a subset of immune- and stress-related genes to gauge which microbes may be associated with the most ‘damage’ to the host, hence potentially impacting performance of wild fish.

### Evolution of microparasites

Microparasites evolve responsive and adaptive molecular traits that enable efficient adherence, entry and replication within the host (Pulkkinen et al. 2010). Virulent microparasite strains have greater infectivity, higher tissue-degrading capacity and higher growth rates but are not generally selectively favored in nature if death of the host limits the population cycle of the microparasite (Pulkkinen et al. 2010). However, infectious agents that can maintain infectivity for months in fresh or seawater or in the soil will endure a lower fitness cost of host death and are thus more likely to undergo selection for increased virulence in natural populations (Pulkkinen et al. 2010).

There is strong empirical evidence that evolution for enhanced microparasite virulence can proceed quickly in a culture environment because local extinction of infectious agents after spikes of disease does not occur if there is no limitation on host animals (Anderson and May 1982; Frank 1996; Ebert and Mangin 1997; Altizer et al. 2003; Murray and Peeler 2005). Continuous introduction of naïve fish to meet production demands, selection of recovered fish, and lack of control methods for novel microparasites all contribute to the evolution of enhanced virulence (Kurath and Winton 2011). Cooccurrence of multiple genetically distinct microparasite strains within the same population will also favor virulence if more virulent strains have a competitive advantage (Nowak and May 1994; Frank 1996; Gandon et al. 2001; Read and Taylor 2001). Moreover, the use of drugs to suppress and kill parasites in cultured fish not only selects for drug resistance, but may also exacerbate selection for faster growth and transmission (Mennerat et al. 2010). Use of vaccines that reduce pathogen growth may also reduce the cost of virulence, selecting for higher virulence due to reduced risks of host death (Mennerat et al. 2010).

RNA viruses are the best examples of rapid evolution of virulence of microparasites in cultured salmon. In salmon, eight RNA viruses are associated with emerging diseases in aquaculture (IHNV, ISAV, IPNV, PMCV, PRV, viral hemorrhagic septicemia virus [VHSV], salmon alphavirus [SAV], Atlantic Salmon paramyxovirus [ASPV]), many of which show evidence of rapid evolution on farms. For example, in farmed Rainbow Trout, genetic analyses of more than a thousand isolates of IHNV show higher levels of genetic diversity, faster rates of evolution, and independent evolutionary trajectories compared to ancestral wild isolates (Troyer et al. 2000). Similarly, VHSV genotype I has undergone rapid evolution in domesticated Rainbow Trout in Europe, producing a number of highly virulent strains (Kurath and Winton 2011). In Norway, only the avirulent ISAV-HPR0 strain has been observed in wild fish, whereas both HPR0 and virulent strains of ISAV are common in salmon net pens (Plarre et al. 2005, 2012). While horizontal transmission has been considered a dominant route of exchange of virulent strains of the virus, a recent study by Plarre et al. (2012) proposed that virulent strains are repeatedly evolving on ocean farms from HPR0 strains common in wild populations.

Increased virulence under culture is not limited to viruses. Virulence of the bacterial pathogen *Flavobacterium columnare* in salmon fingerlings farmed in northern Finland is hypothesized to have evolved from fierce strain competition in high density rearing environments (Pulkkinen et al. 2010). The evolved virulent strains have higher infectivity and growth rates and are associated with increased severity of symptoms prior to death of the host. Moreover, they can transmit from dead fish and remain viable in sterilized water for months (Pulkkinen et al. 2010). Furunculosis has also increased in virulence in cultured fish (Bakke and Harris 1998). In salmon aquaculture, there are attempts to minimize disease outbreaks and the evolution of enhanced virulence by limiting exposure between year-classes and leaving sites fallow after harvest before new fish are introduced (Costelloe et al. 2001). For a more detailed description of parasite and pathogen evolution on salmon farms, see Mennerat et al. (2010).

### Introductions of exotic microparasites

The introduction of novel microparasites may be associated with ‘virgin ground’ epidemics that progress quickly through previously unexposed populations and cause high mortality and striking reductions in host abundance (Altizer et al. 2003). However, to differentiate impacts associated with introduced diseases from those of climate or other factors that may influence population dynamics, abundance data before and after potential introductions are required (Hochachka and Dhondt 2000; Daszak et al. 2005; Lips et al. 2006; LaDeau et al. 2007). As a result, such outbreaks in species or populations that are not closely monitored would likely go undocumented; such is likely the case for wild salmon. The best examples of virgin ground epidemics come from terrestrial systems, with distemper outbreaks in European seals (Jensen et al. 2002), *Mycoplasma gallisepticum* and West Nile virus outbreaks in wild avian populations (Hochachka and Dhondt 2000; LaDeau et al. 2007), and outbreaks of a pathogenic chytrid fungus, *Batrachochytrium dendrobatidis*, threatening amphibian biodiversity in Panama (Lips et al. 2006). Whether new outbreaks are the results of ‘host jumps’ or introductions through natural shifts in carrier distributions due to climate or anthropogenic-associated movements, we expect that if host populations have maintained sufficient diversity, emerging diseases will ultimately be both buffered by and change rapidly the genetic composition of host populations (Altizer et al. 2003). To date, there is more support for emergence from geographic proximity and opportunities for cross-species transmission rather than genetic changes in the infectious agents themselves (Altizer et al. 2003). The best-known example of species cross-over caused by a mutation in the infectious agent is with the relatively benign feline parvovirus. In the 1970s, mutations in the capsid protein of the virus altered the recognition of the host transferrin receptor and caused the virus to be infective and highly virulent in canines, leading to epidemic outbreaks impacting wolves, coyotes and domesticated dogs (Parrish and Kawaoka 2005). Another example is the recent avian epizootics of high-pathogenicity strains of H5N1 influenza A which jumped to mammals and caused small outbreaks and death in humans (Parrish et al. 2008).

In salmon, the homing response, which returns spawning salmon to their natal river, can serve to lessen natural exchange of microparasites between freshwater systems, and osmotic barriers associated with some microparasites would also reduce potential for exchange (Bakke and Harris 1998). These barriers to microparasite movements between freshwater systems would serve to enhance the variance in evolved resistance among populations, consistent with the patterns of MHC variation observed in anadromous salmon. Alternately, we expect that species or stocks that have lower site fidelity for spawning may be exposed to a larger array of microparasites and hence evolve a higher capacity for resistance. As conditions warm, successful colonization in more northerly latitudes may increase (Babaluk et al. 2000), enhancing the dispersal of microparasites among systems. For systems with no evolved resistance, new microbe introductions could result in localized disease outbreaks.

Translocation of microparasites through human activities is also a concern, and there are several documented cases where this has resulted in devastating effects. On a local level, translocation of fishes by anglers or enhancement facilities can introduce microparasites into systems where they were otherwise absent (Bakke and Harris 1998). Escapees from salmon farms are also a potential source of microparasite infections in wild fish, although examples of such occurrences are rare. In Europe, farmed escapees have been blamed for furuncolosis outbreaks in wild fish (Johnsen and Jensen 1994). However, it is the large-scale transfers of fish and eggs that are considered the highest risk toward introduction of nonendemic pathogens. The accumulation of exotic microbes in the Chilean salmon aquaculture industry (6 bacterial, 3 viral and 2 parasitic salmon pathogens; Table 1), which was salmon disease free when the industry started in the early 1990s, is strong evidence of this risk (Ibieta et al. 2011). In Europe, Bakke and Harris (1998) suggest that the most devastating impacts of disease transfer through fish movements has been furunculosis outbreaks that occurred originally during the nineteenth century coincident with movements of juvenile salmonids across the Atlantic and within Europe (Lund 1967), with a second reintroduction occurring more recently across Europe (Egidius 1987). As well, there is some evidence that *G. salaris*, which is endemic and nonpathogenic in Finland, has been introduced through the movement of Rainbow Trout from Finland into Russia (Mo 1994), Germany, Spain, Denmark, and Portugal (Malmberg 1993). Similarly, outbreaks of *M. cerebralis*, the causative agent of whirling, in the United States followed translocations of live Rainbow Trout from Europe, most notably Germany, after WWII (Bartholomew and Reno 2002). While there has been speculation that PRV newly discovered in BC salmon is a result of recent egg imports (Kibenge et al. 2013), there is no compelling evidence to date of diseases impacting wild Pacific salmon in North America that resulted from egg transfers associated with the aquaculture industry. However, there is evidence to support the very high impact that an endemic North American virus, IHNV, has had on the exotic Atlantic Salmon that are the mainstay of the aquaculture industry (Saksida 2006).

### Potential for exchange between wild and cultured salmon

As wild salmon populations in North America and Norway have been declining in both numbers and productivity, aquaculture production has been increasing (Ford and Myers 2008; Walker and Winton 2010). There is growing evidence that in some regions, aquaculture may be a primary cause of declines in wild populations (Ford and Myers 2008). Reductions in fitness due to genetic introgression of farmed escapees (where endemic species are cultured) and transfer of disease are the main issues of concern (Heggberget et al. 1993). Disease exchange from aquaculture to wild fish may occur through the introduction of novel microparasites by translocations of eggs or juvenile fish, or as a result of artificially high carrier states of endemic microparasites due to high density rearing environments (Krkošek et al. 2006). Additionally, net pen farming could increase concentrations of myxozoan parasites by creating optimal environments for their intermediate invertebrate hosts (e.g., annelid worms) in the eutrophic environment under salmon pens (Johnsen et al. 1993), potentially increasing their impact on both farmed and wild migrating populations (Bakke and Harris 1998).

In aquaculture, fish can be reared at densities more than a thousand times those in natural environments (Pulkkinen et al. 2010). A fundamental principle of epidemiology is that populations should be most subject to host-specific infectious disease when they are at high densities (Lafferty and Gerber 2002). This is a key tenet of the premise that populations in a culture environment will be more affected by disease than wild populations; given what we know about disease outbreaks on farms, this does appear to be the case (Ibieta et al. 2011). In the section on microbial evolution above, we discussed the factors in addition to density present in a culture environment that facilitate rapid evolution of enhanced virulence. However, most evidence to date suggests that it is not the highly virulent microparasites produced by high density salmon culture that are the greatest risk to wild populations (Anderson 1979; Bakke and Harris 1998; Biering et al. 2013). For example, molecular monitoring of wild Atlantic Salmon and sea trout (*S. trutta*) in Norway revealed that only one of the five emerging viruses (PRV but not IPNV, SAV, ISAV, or PCMV) impacting the salmon aquaculture industry was present in >1.5% of wild fish, nor were the two most pathogenic bacterial microbes, *R. salmoninarum* and *A*. *salmonicida* present at appreciable levels among the 500 fish surveyed (Biering et al. 2013). These prevalence rates differed dramatically from those associated with the Norwegian aquaculture industry, which had been experiencing particularly high incidence of IPNV and SAV. The question is, did affeced wild fish simply die unsampled or is there really a much lower infection pressure on wild fish (McVicar 1997)?

Studies from terrestrial systems indicate that cultured animals can be important carriers of disease, even if the cultured species suffers little pathology (Lafferty and Gerber 2002). Terrestrial examples of domestic/wild impacts of disease exchange are abundant and have involved bacterial, fungal, viral, and protozoan infectious agents that have reduced wild populations of affected species by 80–90%, occasionally causing local extinction (reviewed in Lafferty and Gerber 2002). In the aquatic realm, a survey from ProMED-mail in 2000 revealed that hatcheries and aquaculture facilities were associated with the North American spread of ISAV and salmon sarcoma virus in Atlantic Salmon, and whirling disease (*M. cerebralis*) and furuncolosis in trout (Dobson and Foufopoulos 2001). In Norway, disease outbreaks of gyrodactyliasis (caused by *G. salaris*) and furunculosis leading to severe declines in wild populations are highly correlated with the expansion of the aquaculture industry in the northwestern Atlantic and the Baltic during the first half of the 1980s (Johnsen and Jensen 1994; Heggberget et al. 1993). The scale of *G. salaris* losses was so great in Norwegian salmon rivers that entire systems were treated with rotenone in an attempt to eradicate the parasite (Windsor and Hutchinson 1990).

Disease transfer between aquaculture and wild populations is not unidirectional; there are several documented cases where disease outbreaks on farms have occurred after transmission of infectious agents from wild fish; in fact, there are more substantiated reports of wild to aquaculture disease transfer than aquaculture to wild (viral transfer reviewed in Kurath and Winton 2011). A case in point in the northeastern Pacific are the widespread outbreaks of IHNV soon after the Atlantic Salmon farming industry was established in the early 1990s (St-Hilaire et al. 2002). As Atlantic Salmon are an exotic species in the Pacific Ocean, they had no natural resistance to microbes endemic to BC salmon. IHNV is endemic to BC and is particularly prevalent in Sockeye Salmon populations in freshwater (Rucker et al. 1953; Traxler et al. 1998; see sections above for more discussion on IHN). From 1992–1996, cumulative mortality from the IHN outbreaks on BC farms was close to 50%, similar to levels experienced during a second outbreak from 2001 to 2003 (Saksida 2006) associated with losses of over 12 million fish. Sequence-level analyses resolved that outbreaks resulted from three separate introductions from viral strains common in wild Pacific salmon populations from Alaska, BC and Washington State (Saksida 2006).

The probability of disease transfer between aquaculture and wild fish in the marine environment will largely depend upon the hydrographic regime around the net pens, the migration routes of wild fish and length of time that wild and farmed fish are in close contact, prevalence of infection, shedding rates, and the longevity of microparasites outside of their host. Models that include detailed field observations and oceanographic mapping to define potential dispersal routes within and between host metapopulations are rare (Bakke and Harris 1998). Research on sea lice dispersal patterns in Europe (Costelloe et al. 1996, 1998) and circulation models around salmon farms in BC to better understand potential dispersal patterns of IHNV and sea lice (Foreman et al. 2012) are the exception. Without this research, the epidemiological consequences of open net pen farms associated with aquaculture, and of movements of juvenile salmon between river systems, cannot be adequately assessed.

The transfer of disease between farmed and wild fish does not necessarily require direct contact between the two populations. Microparasites can also be transported by predatory birds (McAllister and Owens 1992) and fish (Glover et al. 2013), and by escapees from farms (Munro et al. 1976). Avian scavengers may travel long distances, spreading diseases between freshwater and marine habitats (Murray and Peeler 2005); IPNV has been found in the feces of scavenging sea gulls (McAllister and Owens 1992). Predatory wild Atlantic cod (*Gadus morhua*) have also been shown to be carriers of PRV likely originating from nearby salmon farms (Glover et al. 2013).

Direct exchange of microparasites between cultured and wild fish is certainly not the only route of microbe exchange. Many microparasites have intermediate invertebrate hosts; hence environments that foster naturally high densities of intermediate hosts may enhance levels of natural populations. Marine fish, such as herring (*Clupea pallasi*), threespine sticklebacks (*Gasterosteus aculeatus*), Pacific hake (*Merluccius productus),* and Pacific sandlance (*Ammodytes hexapterus*) are routinely cocaptured in aggregations of salmon smolts or in areas around salmon farms, and are known to harbor microparasites that can infect salmon. Salmon microparasites known to be carried by marine fishes include: Viruses— VHSV, ISAV, and IHNV; bacterial microbes—*R*. *salmoninarum*, chlamydia-like organisms*;* microparasites—*Loma* sp. (Nylund et al. 2002; Kent et al. 1998). Sea louse are important salmon macroparasites, and may be important vectors for viruses (e.g. ISAV – Nylund et al. 1993; IHNV – Jakob et al. 2011), bacteria (*A*. *salmonicida* – Nese and Enger 1993) and microparasites (*Paranucleospora theridion* – Freeman and Sommerville 2009; Jones et al. 2012).

### Potential for exchange between hatchery and wild fish

The Salmon Specialist Group of the International Union for the Conservation of Nature (IUCN) listed ‘negative effects of hatcheries and construction of artificial spawning habitat,’ including the spread of disease to wild salmon, as one the their three major threats to Sockeye Salmon (Rand 2008). While there is relatively strong evidence for genetic impacts on fitness (reviewed in Naish et al. 2008), direct evidence for the role of disease is lacking (Stephen et al. 2011). We restrict our brief discussion of hatchery impacts herein to the transmission of disease.

Hatchery fish reared for enhancement are exposed to the same environments as wild fish for the marine phase of their life cycle and freshwater return migration; hence, other than at natal rearing areas (or hatcheries), the same endemic microparasite reservoirs are the source of infectious diseases for both (Naish et al. 2008). However, when infectious diseases occur, the prevalence and intensity of infection may grow faster in a high density hatchery environment than in the wild (Naish et al. 2008). As with aquaculture, hatchery fish may facilitate microparasite transfer through the intentional movement of cultured fish carrying undetected exotic microbes (examples include the spread of whirling disease in the United States and *G. salaris* in Europe discussed previously) and amplification of endemic microparasites in high-density rearing environments (released through untreated hatchery effluents), for which there is limited direct evidence (Naish et al. 2008). Intentional release of infected fish can also occur, but the consequences of these releases on wild fish have not, to date, been monitored (Stephen et al. 2011). Perhaps the best example of this potential is in enhanced disease in hatchery and wild fish barged together to facilitate transport around dams in the Columbia River (Elliott et al. 1997). Alternately, hatcheries that aim for disease free environments through use of well water may release large numbers of susceptible naïve fish to the environment, which may cause localize outbreaks of disease (Naish et al. 2008).

Lack of regular microparasite monitoring in hatchery and wild stocks may largely explain the limited data available to assess disease interactions between hatchery and wild fish (Krueger and May 1991). Disease-monitoring programs vary widely between individual hatcheries (Stephen et al. 2011) but are largely limited to broodstock assessments for a small number of vertically transmitted microparasites [e.g. *R. salmoninarum* and IHNV in the Pacific Northwest (Stephen et al. 2011); *R*. *salmoninarum*, furunculosis, and IPNV in Norway (Biering et al. 2013)]. Veterinary diagnostics may be performed during mortality events. Case reports from BC enhancement hatcheries have identified a wide range of pathogens, including viral (3), bacterial (7), microsporidian (2), myxozoan (2), protozoan (2), ameba (1), ciliate (1), and an ectoparasitic worm (1) infecting salmon in BC hatcheries (Table 1).

### Climate change shifts the balance

There is an increasing concern about the potential ways in which global warming or climate change can alter the severity or distribution of diseases affecting aquatic animals (Harvell et al. 1999; 2002; Lafferty et al. 2004; Marcogliese 2008; Echaubard and Lesbarrères this issue). Most infectious agents have short generation times and large population sizes. Moreover, strong selection following ecological changes, like those associated with shifts in climate, might accelerate pathogen evolution (Altizer et al. 2003). Latitudinal diversity gradients for pathogen richness track those of general species diversity increasing from the poles to the equator (Rohde and Heap 1998; Rohde 1999; Guernier et al. 2004), with temperature a contributing factor generating this variation (Clarke and Gaston 2006). Retreat of perennial sea ice has shown acceleration in recent decades (Comiso et al. 2008); subsequently increased Arctic passage has the potential to promote range expansion of various marine species and their associated pathogens across ocean basins (Post et al. 2013). A comprehensive understanding of host/pathogen relationships and their nuances among species, populations and life stages (e.g., salmonids) is critical to anticipating region-specific impacts on disease potential within the context of spatially varying climate-related changes in associated abiotic factors (e.g., temperature; Altizer et al. 2013).

It has been well established that temperature is a critical environmental factor that affects the progression of disease in fish (Wedemeyer 1996). High water temperature (HWT) can affect disease progression through direct effects on host physiology that compromise immune system function or direct effects on microparasites that alter their replication rate (Noe and Dickerson 1995; Marcogliese 2001), likely involving both plastic and evolutionary mechanisms. Temperature increases can also impact development rate and timing of release of microparasites from intermediate hosts, potentially increasing densities and and extending exposure periods for migratory fish (Stocking et al. 2006; Ray et al. 2012; Chiaramonte 2013). Hence, migration timing, often associated with river temperature, is an important aspect impacting disease potential, especially via microparasites with intermediate hosts (e.g. *C. shasta*, *P*. *minibicornis*).

HWT has been identified as a source of stress especially during crucial life-history stages such as adult spawning migration (Crossin et al. 2008; Eliason et al. 2011, 2013a; Clark et al. 2012) and is a primary factor affecting adult survival relating to overall fitness (Martins et al. 2011, 2012a,b). Thermal tolerance has been classified by several studies as species- or population-specific, consistent with historic temperatures (Lee et al. 2003; Farrell et al. 2008; Clark et al. 2011; Eliason et al. 2011, 2013b), and likely the result of selection (Crozier et al. 2008). Studies of handling stress at elevated temperature across a wide range of species have shown that deleterious effects occur within the bounds of a preferred temperature range rather than above (Gale et al. 2013); hence, additional stressors such as microparasite infection could have enhanced impacts at even slight temperature increases. Water temperatures above the thermal optimum could adversely affect swimming stamina of naturally migrating fish or fish evading predators and fishing nets in the river, regardless of infection status (Farrell et al. 2008). Sustained swim performance is substantially inhibited between 18 to 21°C, above which fish can no longer maintain homeostasis and is immediately lethal (Farrell et al. 2009). Such inhibition is supported by observed migration failure of wild stocks when river temperatures exceed 18°C (Crossin et al. 2008; Jeffries et al. 2012; Keefer et al. 2008; Martins et al. 2011, 2012a). Proposed mechanisms contributing to decreased stamina and migration failure in the presence of HWTs include increased energy use (Rand et al. 2006), decreased dissolved oxygen (Eliason et al. 2011), as well as severe microparasite infections resulting in lower critical swim speeds and longer recovery rates. (Tierney and Farrell 2004; Wagner et al. 2005; Kocan et al. 2009). Decreased swim performance arising from infection could increase exposure time to HWT and vulnerability to predation, further exacerbating the potential for cumulative impacts.

If differences in microparasite virulence under HWTs result from reduced condition of the host (i.e., a weaker immune response), then evolutionary variance in susceptibility to temperature stress may play a larger role than plastic responses in pathogen-temperature outcomes, with predicted greater tolerance to microparasites in fish with greater resistance to HWT stress. Hence, direct effects of temperature on pathogen virulence may manifest differently among populations depending on evolved variances in temperature susceptibility; animals that are not stressed directly by high water temperatures may be more refractory to pathogens showing enhanced virulence with temperature. Moreover, the degree of energy allocation to the immune response may be pathogen- and host-dependent at HWT, as exemplified in the relatively stronger transcriptional response of Atlantic cod to viral versus bacterial pathogens under HWT conditions (Hori et al. 2013).

Cumulative chronic and acute stressors impacting salmon stocks are in need of quantitative evaluation using a multivariate approach (Johnson et al. 2012) and evolutionary perspective to anticipate population-specific variability in HWT responses of host and pathogen. A large-scale multispecies evaluation of disease potential in adult salmon during spawning migration and in response to both thermal and fisheries stressors has produced preliminary findings presented in our third case study described below. Using a microparasite screening approach of wild fish collected and held in a laboratory setting, we manipulated temperature during a simulated migration to monitor differences in microbe load and associated mortality trends between temperature treatments.

### Predators—the ultimate cause of death of infected wild fish?

During their marine life, Pacific salmon experience variably heavy mortality rates that generally exceed 90% (Bradford 1995). Mortality arising from nonanthropomorphic predation is thought to be less common in homeward migrating fish upon river entry, but still can occur from marine mammals and bears (Quinn and Kinnison 1999), and may be mediated by other stressors like fisheries interactions (i.e., postrelease predation of discards by seals; Donaldson et al. 2011). The losses in the marine environment are thought to be caused primarily by predation in the first few weeks to months following ocean entry, and by weakened condition due to food limitation during the first winter at sea (Beamish and Mahnken 2001). While mortality from both causes is thought to be size- and condition-dependent (Willette 2001; Hurst 2007), the supporting evidence tends to be indirect and inferential. For example, size-selective mortality is typically inferred from reconstructions of fish lengths from recovered hard parts (scales and otoliths) (Healy 1982), but this method precludes an assessment of variation in body mass, condition or health, and rarely are characteristics of survivors and nonsurvivors compared simultaneously. Although size-selective survival for salmon is commonly recognized (e.g., Saloniemi et al. 2004), at times it is not observed (Welch et al. 2011) or the effect is negative (Ewing and Ewing 2002). Nonetheless, conditions that lead to decreased growth and energy storage are expected to increase mortality rates and ultimately decrease returns of adult salmon (Beamish et al. 2004). For example, environmental conditions that lessen the quality and availability of food can decrease growth rates resulting in poor physical condition (Tocher 2010; Duffy and Beauchamp 2011; Tomaro et al. 2012). Poor physical condition can reduce salmon health and survival directly through immune suppression and susceptibility to pathogens (Peters et al. 1988; Arkoosh et al. 2006). Poor body condition has also been linked to a reduction in the capacity of fish to evade predators under controlled conditions (reviewed in Mesa et al. 1994), and in river environments (Hostetter 2009). However, the actual sources of mortality let alone the role of body condition and other indices of the health of juvenile salmon in determining their susceptibility to predators while at sea remain a black box given the difficulty of studying fish in such a dynamic environment over enormous geographic scales ultimately facing an array of potential predators. This becomes even more confounding given the likely potential for conflating interactions between environmental conditions, competition, disease, and predation.

Selection of prey in poor body condition is a widespread phenomenon in terrestrial systems (Murray 2002; Husseman et al. 2003). The tendency for terrestrial predators to take substandard prey is linked to hunting strategy where predators that pursue their prey are more likely to take individuals in poorer condition compared to those with ambush tactics given impeded escape ability and/or state-dependent risk taking (Fitzgibbon and Fanshawe 1989). Similar patterns of prey selection are often assumed to operate for salmon in the ocean (Burke et al. 2013). A laboratory study by Mesa et al. (1998) demonstrated that Chinook Salmon challenged with *R*. *salmoninarum* were more susceptible to predation by Northern Squawfish (*Ptychocheilus oregonensis*) and Smallmouth Bass (*Micropterus dolomieu*) under experimental conditions. We found a single field study that assessed the impact of condition and microparasites on predation in wild salmon (Hostetter 2009). They documented external condition characteristics (e.g., body injuries, descaling, external signs of disease, fin damage, and ectoparasite infestations) of tagged out-migrating Steelhead Trout smolts in the Columbia River and noted that recoveries of tags at downstream colonies of Caspian Terns (*Hydroprogne caspia*) and Double-crested Cormorants (*Phalacrocorax auritus*) not only indicated that smaller Steelhead were taken but that predation was highest on Steelhead displaying signs of poor condition. Moreover, external condition was correlated with the presence of selected pathogens detected by histopathology and molecular analysis. While the indices of condition were somewhat qualitative and the suite of pathogens restricted, results are intriguing.

In general, condition-based susceptibility and the role of disease in the marine environment remains untested given that predator/prey interactions are difficult, if not often impossible to observe. In case study IV, we identify one predator/prey system that is amenable to observation and direct testing of the condition-based predation hypothesis. The Rhinoceros Auklet *Cerorhinca monocerata* is an abundant, pursuit-diving seabird that consumes copious quantities of salmon post-smolts, delivering them whole and intact to nestlings (Thayer et al. 2008). During migration, the vast majority of juvenile salmon from southern and central BC stocks funnel past aggregations of hundreds of thousands of auklets that breed on colonies scattered along BC’s Central and North coast (Tucker et al. 2009, 2012). We were able to collect freshly caught smolts from auklet nests and contrast their condition and infection status with that of smolts in the general population. Although the scale of the study was small, it is one of the few studies able to make direct contrasts between predated and unpredated salmon in the field.

## Perspective on moving forward

Establishing a direct cause and effect relationship between pathogens and disease may not be possible in wild populations if pathogenicity of an infectious agent causes infected fish to die and disappear before they are detected (Bakke and Harris 1998). Hence, to understand the role of infectious diseases on wild salmon, it is important that we merge both field studies that allow for the discovery of factors associated with survival in complex natural environments with controlled laboratory studies that can test hypotheses gained from field studies and provide a stronger mechanistic basis to findings. There is a strong foundation of research on distributions and impacts of salmon macroparasites in wild salmon, largely because these are readily observable either to the naked eye or using microscopy (Margolis and Arthur 1979; McDonald and Margolis 1995; Bennett et al. 1998; Kent et al. 1998; Arkoosh et al. 2004; Ferguson et al. 2012). Microparasites have received much less focus in wild fish, despite the fact that they have caused the most devastating impacts on cultured fish. Bacterial kidney disease (BKD), vibriosis, ceratomyxosis, and enteric redmouth have, however, been observed in wild migrating fish (Arkoosh et al. 2004; Kent et al. 1998; Fujiwara et al. 2011; Rhodes et al. 2011). A small number of studies that have conducted sequential sampling have used overdispersion (mean to variance ratios) of parasites as indirect evidence of mortality (Gordon and Rau 1982; Kalbe et al. 2002; Jacobson et al. 2008). Alternately, use of negative binomial distributions truncation technique described by Crofton (1971) has been a widely accepted model for macroparasites (see Scott and Smith 1994; Ferguson et al. 2011).

Traditional diagnostic approaches relying on observed mortality events are not sufficient to study disease in natural systems. The probability of finding near-moribund-infected fish in random samples of wild-caught salmon is low, and damage at the cellular level that characterizes different types of diseases may be difficult to resolve with histology. Because successful cell culture generally requires a moderate load of viruses or bacteria (Templeton et al. 2004), culture may additionally miss detection of animals at an early stage of infection. Moreover, culture-based methods may underestimate microparasite presence, as all microparasites are not cultivable (e.g., PRV, PMCV, ISA-HPR0, others). ELISA’s can be an effective diagnostic method to identify well-characterized infectious agents but are not generally as sensitive as molecular approaches (Sandell and Jacobson 2011). Quantitative RT-PCR is generally the most sensitive method to detect presence and load of microparasites (Purcell et al. 2011), but in some instances may not be as sensitive as culture-based methods for diagnosing disease, as it is unable to determine whether a microbe present in a tissue is viable and actively replicating (Purcell et al. 2013).

The fact is that we have not adequately characterized the range of microparasites that wild salmon carry, especially in the marine environment. Most of the recently discovered microparasites associated with emerging diseases in Europe have not even been assessed for the presence in North America. Hence, we, along with numerous other scientists studying wildlife populations (Bakke and Harris 1998; Dobson and Foufopoulos 2001), argue that a broad characterization of the microparasites carried in the wild would provide a good foundation to research aimed at establishing the role of infectious disease in natural systems.

### BC salmon health initiative

We have developed a multidisciplinary research program, the Strategic BC Salmon Health Initiative (SSHI; http://www.genomebc.ca/portfolio/projects/fisheries-projects/strategic-salmon-health-initiative/) that merges the fields of genomics, epidemiology, histopathology, virology, parasitology, fish health, veterinary diagnostics, and salmon ecology to assess the potential role of infectious disease as a cofactor in wild salmon declines. The core of this research is the evaluation and application of a high-throughput microfluidics platform for the identification and quantification of important viral, bacterial, protozoan, and fungal microparasites that may influence the health and survival of native populations of BC salmon. Using this technology, the research will characterize broadly the range of microparasites carried by wild salmon, assess variance in diversity and loads of microparasites carried in populations of wild and cultured salmon during smolt out-migration and adult return-migration, assess genetic variance in host response to specific microparasites, conduct association studies between microparasites, host immune genes, and fate (using biotelemetry), and assess in experimental studies which microparasites are further stimulated to replicate under elevated temperatures and handling stress (catch/release fisheries). This program is also integrating histopathology to identify lesions associated with cellular damage that may be associated with high loads of specific microparasites, important to begin to link microparasite carrier states with potential for disease. Epidemiology studies will incorporate full viral genome sequencing to characterize the distribution and potential for exchange of viral microparasites of interest. NGS will also be used for viral discovery research. Laboratory challenges of understudied microparasites that carry the greatest potential for population-level impacts (i.e., of sufficient prevalence and load, possibly with evidence of shifting prevalence/load over migration, and associated with strong genomic responses and evidence of cellular damage) will follow to determine cause and effect relationships between microparasites and disease and to determine under what conditions disease occurs. Ultimately, if microparasites potentially associated with salmon productivity are identified, studies that provide the evolutionary framework upon which disease ensues—based on genetic variation in microparasites and host—will be pursued.

To effectively tackle cumulative impacts of multiple stressors, we are clearly going to need to employ modern, sophisticated tools, and approaches for studies conducted in natural systems. Ideally, these would merge molecular-based monitoring tools, genetic markers to differentiate populations, gene expression profiling to assess condition and health, biotelemetry to relate biological and physiological metrics of condition and health with shifts in behavior and fate, and oceanographic data to incorporate abiotic factors. Herein, we present a series of three ‘proof of concept’ field studies and one laboratory study that utilize a combination of novel approaches to explore the range of microparasites potentially impacting wild salmon populations and the cumulative impacts of genetics, temperature stress and predators on the diversity and loads of microparasites and ultimate disease outcomes. In future, the intent is to merge these approaches with full-genome scans (i.e., QTL discovery and full parental genotyping of hatchery fish) that will provide a greater mechanistic understanding of the evolutionary impacts of cumulative stressors on wild salmon populations.

The highlighted studies were developed to test a number of null ecological and evolutionary hypotheses, including (i) there are no genetic differences in the diversity, range and load of microparasites carried by wild salmon populations that have reared in a common ocean environment (adult liver study), (ii) microparasite carrier states are not predictive of migratory fate of return-migrating salmon, and if they were, there are no population-specific differences in microparasite associations (2010 tracking study), (iii) temperature and handling stress do not impact microparasite replication or virulence, or subsequent survival of salmon (Coho handling study), and (iv) there is no association between salmon infection status and seabird predation (Auklet study).

### Foundations of the novel and merging of technologies presented in our studies

Molecular technologies are rapidly changing the ways we approach ecological and evolutionary research and the depth of information that can be gained both quickly and relatively cheaply. Common and emerging applications, with examples in aquatic/salmon biology, include:

1. Genetic assessments, which when based on a small number of markers (e.g. microsatellites or SNPs) have been used to identify population compositions of mixed population samples of Pacific salmon—routinely applied in salmon management and to identify population-specific migration routes (Beacham et al. 2008; Tucker et al. 2009, 2012), and to identify population of origin of individuals—used to assess the performance and condition of different stocks groups across diverse habitats (Cooke et al. 2008; Miller et al. 2011; Hinch et al. 2012). Genome-scale genetic assessments (e.g., fully mapped microsatellites, RAD-tag sequencing) have been used in QTL discovery and to identify adaptive genetic variation among populations (Houston et al. 2008; Miller et al. 2012). Herein, we apply genetic population identification based on microsatellite loci and SNPs on wild-caught individuals to assess (i) the relative impact of genetic variation (at the population level) in microparasite diversity and load, and (ii) to determine the importance of genetic variation (at the population level) in microparasite associations with migratory fate.
2. Gene expression profiling to elucidate response to stressors, based on both targeted gene ‘biomarker’ approach (e.g. qRT-PCR of biomarkers known to associate with disease, stress, environmental adaptation; Elder et al. 2008) and genome-based approaches [e.g., microarrays assessing the activity of 10s of thousands of genes or NGS of RNA transcripts (RNA-Seq) (Salem et al. 2012)]. Herein, we employ a targeted gene approach alongside the microparasite monitoring applied on a microfluidics platform.
3. Monitoring systems to determine the presence and relative abundance/load of species/strains of interest (e.g., microparasites, harmful algal bloom species, planktonic communities, gut contents, and invasive species). Research on microbial communities is perhaps the farthest along when it comes to large-scale molecular-based monitoring, with NGS approaches used to simultaneously identify species compositions and functional trajectories of common-place microbial communities (MacLean et al. 2009). Molecular virology has also utilized a NGS approach to discover viruses that control phytoplankton bloom cycles (Suttle 2007). However, research and monitoring of infectious agents is far behind, largely employing single assays as a diagnostic tool to assess potential associations of a small number of microparasites with disease and mortality. Herein, we expand on this approach and present studies that utilize a microfluidics qRT-PCR platform that can simultaneously run 96 TaqMan assays on 96 samples (Fluidigm BioMark™, Fluidigm Corp., San Francisco, CA, USA). This system has similarly been used in microbial water monitoring (Ishii et al. 2013). We apply this system for the first time to monitor the presence and load of up to 45 salmon microparasites and verified the key findings of a subset of microparasites on the commonly used ABI 7900 platform.

#### Biotelemetry

The field of biotelemetry has been used effectively to track migratory pathways of a large range of organisms (Ropert-Coudert and Wilson 2005); in the marine realm, sharks, marine mammals, salmon (Rechisky et al. 2013), and tuna have commonly been studied. In 2003, we began merging biotelemetry with nondestructive biopsy sampling of blood and gill tissue from adult salmon (see Cooke et al. 2005 for details on the technique) to determine whether there were associations between indices of physiological condition and migratory behavior and fate (reviewed in Cooke et al. 2008 and Hinch et al. 2012). Over multiple years’ study, timing of river entry and migratory fate were found to be associated with osmoregulation and stress in return-migrating Sockeye Salmon (Cooke et al. 2006; Crossin et al. 2007, 2009; Donaldson et al. 2010). In 2006, we expanded the physiological component of this research to include functional genomics (Miller et al. 2011). The functional genomics study identified a single mortality-related signature (MRS) associated with premature mortality in the river no matter if salmon were tagged in the marine environment, the lower river, or at spawning grounds, providing strong evidence that the condition of salmon in the marine environment impacted the success of migration in the river. Based on the biological processes stimulated within the MRS, we hypothesized that this signature was associated with a response to viral infection. Case study II was a further expansion on this approach, merging molecular monitoring of microparasites and host genes associated with immunity and stress with biotelemetry to explore the linkages between microparasite carrier states, salmon condition, and migratory fate of wild-caught Sockeye Salmon returning to spawn in the Fraser River in 2010.

## Case studies

### Overview

In the following case studies, we assessed diversity and load of a suite of microparasites and conducted association analyses to determine both the factors that explain variations in microparasite distributions (case studies I–III) and the impact of microparasite carrier states on the fate of wild migrating salmon (case studies II and IV). Note that we did not directly assess ‘disease state’ as defined by levels of cellular damage, and we did not attempt to culture microparasites to determine whether they were viable. We did, however, merge host gene expression analysis in case study II to assess which microbes are eliciting a strong response in the host. Given that the microarray studies reviewed above universally show that intensity of host transcriptional response is highly correlated with susceptibility and disease, we use these data to assess which microbes carry the greatest potential for disease at the time the fish were sampled. Future studies will merge histopathology and gene expression analysis with molecular monitoring to identify whether pathology at the molecular and cellular levels is associated with high-load carrier states of microparasites.

### Methods

#### Fluidigm BioMark

In all studies, we conducted qRT-PCR of microparasites, and in some cases, host genes using TaqMan assays run on the Fluidigm BioMark™ platform. We focus largely on microparasites known or suspected to associate with diseases in salmon worldwide (Table 2). Some of the microparasites on our panel are known endemics to BC, others are known to be present in other species but not previously assessed in the species of focus, are recently identified in BC salmon but not extensively studied, or are associated with emerging diseases in Europe but not previously assessed in northeastern Pacific salmon populations (Table 1). Most microparasite assays were from the literature, although a small number were designed in house with Primer Express 3.0.1 software (Life technology, Burlington, ON). Herein, we present results for microbe assays that show strong correlations between the BioMark™ and ABI 7900 platforms, that have been sequence confirmed to verify that the assay is picking up the intended microbe, and that with few exceptions, carry efficiencies above 85%. A full evaluation of the platform performance and impacts on assay sensitivity and specificity for each assay is underway in phase 2a of the SSHI.

**Table 2:** Overview of the microparasites included in case studies

				Prevalence over Case Studies			
Microbe	Agent	Literature Citation	Efficiency	I	II	III	IV
*Aeromonas hydrophila*	Bacterium	Lee et al. (2006)	0.83	N/A	–	–	–
*Aeromonas salmonicida*	Bacterium	Keeling et al. (2013)	0.93	N/A	–	–	–
*Flavobacterium psychrophilum*	Bacterium	Duesund et al. (2010)	0.97	19% *	38%*	–	1%
*Piscichlamydia salmonis*	Bacterium	Nylund et al. (2008)	0.77	–	–	–	–
*Piscirickettsia salmonis*	Bacterium	Corbeil et al. (2003)	0.97	N/A	<1%	–	–
*Renibacterium salmoninarum*	Bacterium	Suzuki and Sakai (2007)	0.94	N/A	–	–	–
*Rickettsia* -Like Organism (Strawberry disease)	Bacterium	Lloyd et al. (2011)	0.94	N/A	4%*	71%	–
Salmon (Gill) chlamydia	Bacterium	Duesund et al. (2010)	0.88	–	3%		4%
*Vibrio anguillarum*	Bacterium	MGL	N/A	N/A	–	–	–
*Vibrio salmonicida*	Bacterium	MGL	N/A	N/A	N/A	N/A	–
*Yersinia ruckeri*	Bacterium	Glenn et al. (2011)	0.98	N/A	–	–	–
Atlantic salmon paramyxovirus	Virus	Nylund et al. (2008)	0.92	N/A	–	–	–
Erythrocytic necrosis virus	Virus	J. Winton (pers. comm.)	N/A	N/A	–	N/A	–
Infectious hematopoietic necrosis virus	Virus	Purcell et al. (2006)	0.97	<1%	1%	–	–
Infectious pancreatic necrosis virus	Virus	S. Clouthier (pers. comm.)	0.97	N/A	–	–	–
Pacific salmon parvovirus	Virus	MGL	0.96	27% *	–	–	23%
Piscine reovirus	Virus	Wiik-Nielsen et al. (2011)	0.90	<1%	19%*	–	–
Salmon alphavirus 1, 2, and 3 (PD/SD/HSS)	Virus	Andersen et al. (2007)	0.91	N/A	–	–	–
Salmonid herpesvirus/Oncorhynchus Masou Herpes Virus	Virus	MGL	N/A	N/A	N/A	N/A	–
Viral encephalopathy and retinopathy virus	Virus	Korsnes et al. (2005)	0.90	<1%	<1%	–	–
Viral hemorrhagic septicemia virus	Virus	Jonstrup et al. (2013)	0.88	N/A	–	–	–
*Gyrodactylus salaris*	Ectoparasitic worm	Collins et al. (2010)	0.89	N/A	–	–	–
*Ichthyophthirius multifiliis*	Ciliate	MGL	0.91	N/A	14%*	98%*	1%
*Nanophyetus salmincola*	Fluke	MGL	0.80	N/A	–	–	–
*Spironucleus salmonicida*	Flagellate	MGL	0.98	N/A	–	–	–
Paranucleospora theridion (syn. Desmozoon lepeophtherii)	Microsporidium	Nylund et al. (2010)	0.78	<1%	19%	–	28%
*Facilispora margolisi*	Microsporidium	MGL	0.83	N/A	1%	–	–
*Loma salmonae*	Microsporidium	MGL	N/A	N/A	32%*	N/A	1%
*Nucleospora salmonis*	Microsporidium	Foltz et al. (2009)	0.99	18%	30%	10%	–
*Ceratomyxa shasta*	Myxozoan	Hallett and Bartholomew (2006)	0.97	N/A	20%*	100%*	1%
*Kudoa thyrsites*	Myxozoan	Funk et al. (2007)	0.90	<1%	–	54%	–
*Myxobolus arcticus*	Myxozoan	MGL	0.96	N/A	<1%	–	2%
*Myxobolus cerebralis*	Myxozoan	Kelley et al. (2004)	0.89	N/A	–	–	–
*Parvicapsula kabatai*	Myxozoan	MGL	0.96	N/A	–	N/A	12%*
*Parvicapsula minibicornis*	Myxozoan	Hallett and Bartholomew (2009)	0.98	N/A	34%*	100%*	35%*
*Parvicapsula pseudobranchicola*	Myxozoan	Jørgensen et al. (2011)	1.29	<1% *	3%	–	7%*
*Tetracapsuloides bryosalmonae*	Myxozoan	Bettge et al. (2009)	0.91	N/A	1%*	38%	N/A
*Cryptobia salmositica*	Protozoan	MGL	N/A	N/A	N/A	N/A	–
*Ichthyophonus hoferi*	Protozoan	MGL	0.88	N/A	1%	2%	5%
*Sphaerothecum destruens*	Protozoan	MGL	0.82	N/A	–	2%	2%

Case studies I and II assessed ocean, river, and spawning ground adult Sockeye, using liver (I) or gill (II) tissue. Case study III surveyed mixed tissues from adult freshwater migrating Coho. In case study IV, liver and gill tissue from ocean-migrating Sockeye post-smolts was assessed. The null 0% prevalence noted as (–), assays not assessed within the case study noted as N/A, significant microparasite marked as (*). Prevalence values presented from case study III include only held fish. Primers obtained from publication are noted with literature citation (MGL primers subject to request). References cited in the Table but not referred to in the text are presented in Reference S1.

The Fluidigm BioMark™ microfluidics platform can run 96 assays against 96 samples at once (9216 reactions on a single dynamic array). As our microparasite TaqMan assays are run in duplicate, we ran up to 45 unique assays and 2–3 housekeeping gene controls per run. We followed manufacturer instructions on the temperature and cycle conditions. Technical details for RNA and cDNA preparation are in Miller et al. 2011 and for the Fluidigm BioMark™ are presented in Data S1.

In each study, tissues were collected in the field and preserved in RNAlater (Qiagen, MD, USA) for 24 hours at 4°C and then frozen in −80°C. In destructively sampled fish, gill, whole brain, liver, head kidney, white muscle, and heart tissues were sampled in the field, whereas in nondestructively sampled fish, only gill was taken. The tissues utilized for microbe monitoring varied by study, as outlined below. Note that in this broad screening, the tissue assessed may not be the primary infective tissue for all microbes monitored; hence, in studies only assessing single tissues (e.g., case studies I and II), we may be underestimating overall microbe carrier states. Individual genetic population identification was performed for all Sockeye Salmon studies on all samples except those collected at spawning grounds (Beacham et al. 2005).

QRT-PCR results were exported as a heatmap csv file and imported into GenEx (www.multid.se) for data preparation and statistical analysis. Data from multiple arrays were combined within GenEx and the average of the duplicated samples calculated. Samples amplifying products from only one duplicate were treated as negative; negatives were all given a threshold cycle (CT) of 50. We used a conservative cut-off of CT<27 to score individuals as ‘positive’ or ‘detected’ for the calculation of prevalence; this equates to a CT of 35–36 on the ABI 7900 and is near the upper limit of reliable repeatability on the ABI instrument. Pearson correlation tests and principal components analysis (PCA) were performed in Genex. Multivariate analyses of variance (manovas) were applied with a randomization procedure (Efron and Tibshirani 1993) in R (R v. 2.15.3; R Development Core Team 2008) to generate test statistics for main effects and interactions in pairwise comparisons. For each analysis, factor labels were randomly permuted 10 000 times to build a permutation distribution rather than compare test statistics to normal distributions. Significance levels were then computed by determining the number of times the reference distribution gave a test statistic equal to or greater than the observed value. If the overall test was significant, pairwise *post hoc* tests were applied to determine which microbes were driving the differences observed. *Post hoc* univariate and multivariate t-tests were also compared with the permutation distributions to determine where the significant differences occurred. Bonferroni corrections were conducted to minimize Type II errors when performing multiple tests; only results significant after correction are reported. The impact of microparasite diversity (count of all detected microbes per individual) and load (count of microbes with CT<20) were additionally explored in some studies using nonparametric Mann–Whitney *U*-test or Chi Square statistics, respectively.

Data preparation and analysis of host genes was also performed in GenEx Enterprise (www.multid.se), in which duplicates were averaged, missing values were filled with column mean, values were corrected for PCR efficiencies (from serial dilutions run), data were normalized (delta-CT) with three reference genes (78d16.1, MrpL40 and Coil-P84), and values were converted to relative quantities with pooled sample data (delta-delta CT), and log_2_ transformed. To determine whether there was an association of host gene expression with specific microparasites, a nonparametric Mann–Whitney *U*-test was performed, with a threshold value of 0.00088 to keep the overall risk of type I error at 0.05 under multiple testing.

### Results

#### Case study I

##### Are the prevalence, load, and diversity of microparasites correlated with host stock, environment, and/or year?

Case study I was a preliminary assessment of the microparasites in liver tissue of return-migrating Sockeye Salmon. A total of 758 Sockeye Salmon were collected from 2005 through 2010 (6 years) from test fishery ocean trawlers in the marine environment (Johnstone Strait, Juan de Fuca Strait), freshwater trawlers in the lower Fraser River, and by beach seine or netting at spawning grounds (as per Miller et al. 2009; experimental design in Table S2). Sockeye have a strong cyclic abundance pattern within populations, and hence, it was not possible to sample all populations in all years (population*year could not be evaluated).

**Figure 1:**
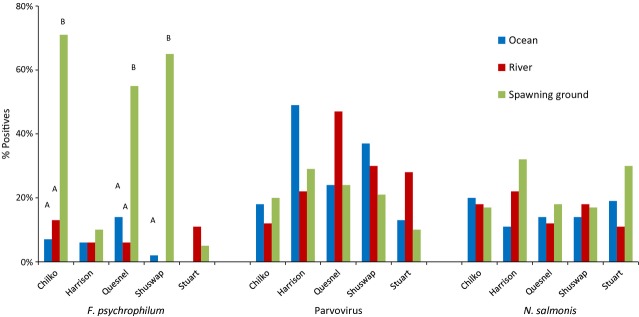
Distribution of *Flavobacterium psychrophilum*, Pacific salmon parvovirus and *Nucleospora salmonis* among stocks and environments in liver tissues of return-migrating Sockeye Salmon (case study I). Environment explained the highest source of variation, with increasing prevalence at spawning grounds in three (Chilko, Quesnel, Shuswap) of the five stocks. Microbes that were significantly different between environments within each stock are indicated by differing letters (i.e., A and B; *P* < 0.001, 1-way manova).

Individual microparasite prevalence over all samples ranged from 0–27%, with six of the 11 microbes surveyed amplifying products with CT<27 in at least two samples (Table 2). Most detected microparasites were present in at least some fish before freshwater entry with the exception of *Kudoa thyrsites*, Gill chlamydia and *P. salmonis*. The three most prevalent microparasites were bacterium *F. psychrophilum*, parvovirus, and microsporidian *Nucleospora salmonis* (Fig. 1). Two-way manovas revealed that environment (*P* < 1 × 10^−4^ in both comparisons) and population (*P* < 1 × 10^−4^ in stock comparison) were the main contributors to the overall microparasite variation. However, an interaction term was also significant between environment and stock (*P* < 1 × 10^−3^). Individuals carried one to three microbes, and with the exception of three of four viruses surveyed (parvovirus, PRV, Viral encephalopathy and retinopathy [VER]), the environmental trend showed enhanced overall microbe prevalence, diversity, and load toward the spawning grounds (Fig. 1).

**Figure 2:**
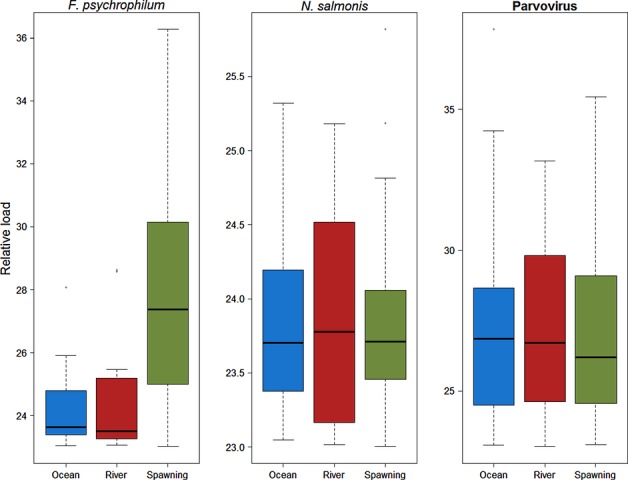
Box plots contrasting the distributions of relative loads (50 – CT) of three microparasites (*Flavobacterium psychrophilum, Nucleospora salmonis*, and Parvovirus) among adult Sockeye Salmon over three environments (ocean, river, and spawning grounds) (case study I). Only samples with detections were used in the calculation of relative loads.

*Flavobacterium psychrophilum* prevalence increased toward the spawning grounds for Chilko, Quesnel and Shuswap populations (Fig. 1). This bacterium is the causative agent of bacterial coldwater disease and is a freshwater pathogen mostly known for its impact on Rainbow Trout fry (reviewed in Starliper 2011); it has not previously been assessed in Sockeye Salmon, and was considered to be of low risk by Kent (2011) due to lack of evidence that Sockeye Salmon were susceptible. An increase in load was observed at the spawning grounds (Fig. 2), suggesting that the bacterium was actively replicating and being transmitted among individuals during migration in the river.

The myxozoan parasite *Parvicapsula pseudobranchicola* was only observed in a small number of fish in 2005 almost exclusively in the Quesnel population (data not shown). While the myxozoan was present in two of three environments (ocean and spawning grounds), the lowest CT was 22, indicating only a moderate load (~10^2^ copies). *P*. *pseudobranchicola* is considered a marine parasite primarily infecting gill tissue, and has been associated with mortalities on salmon farms in Norway (Karlsbakk et al. 2002). There are no data to indicate its pathogenicity in freshwater, and no previous studies in wild fish, or studies documenting its presence in BC (but see also case study IV). The presence of this parasite in BC has been confirmed through sequencing (data not shown).

The Pacific salmon parvovirus, recently discovered in Sockeye Salmon through NGS (K. M. Miller, unpublished data), was highly prevalent in return-migrating Sockeye Salmon, with its distribution more variable by population than environment. Parvovirus is a DNA virus, and these data were based on cDNA, hence we were monitoring the active production of viral transcripts rather than merely the presence of the virus. Parvovirus was present in all years and was in relatively lower prevalence in Chilko (overall prevalence of 16% vs >34% in Harrison; Fig. 1). There was a trend toward lower prevalence at the spawning grounds for four of the five stocks (all but Chilko; Fig. 1). Twenty-one percent of fish amplifying parvovirus carried CT’s <20 (>10^2^ copies per well), with high-load samples distributed across all environments and stocks (Fig. 2). It has not been determined yet whether parvovirus can cause disease in salmon, but it is capable of transmission (K. M. Miller, unpublished data). However, due to enhanced immunosuppression of salmon during their spawning migration, we do not expect that they recover from infections; hence, it is possible that the consistent decreased prevalence toward the spawning grounds is associated with mortality, either directly or indirectly associated with parvovirus infection.

Microsporidian *N. salmonis* was the third most prevalent microparasite (18% over all samples), but did not show a differential distribution based on environment, year or population (Fig. 1). *N. salmonis* is considered by many to be the etiological agent of marine anemia, a disease that has been associated with mortality in Chinook Salmon and Rainbow Trout in the northeastern Pacific (Kent 2011). While this parasite appears to be fairly ubiquitous in adult Sockeye Salmon, it was not observed at high load (CT<20) in any samples (Fig. 2). The high prevalence, low variability, and low load are indicative of a carrier state of this parasite in return-migrating salmon.

#### Case study II

##### Are there microparasites associated with migration success of salmon returning to spawn in freshwater?

In case study II, we assessed whether microparasites already carried by salmon in the marine environment may be associated with premature migration mortality of return-migrating adult Sockeye Salmon in the marine and freshwater environment. Analyses were performed on nondestructively sampled gill tissue collected in the summer of 2010 from fish tagged with acoustic- or radio-tags in the marine environment on the approach to the Fraser River [approximately 100–200 km from the river; see Crossin et al. (2009) and Miller et al. (2011) for tagging and sampling details]. Destructively sampled gill tissue from the lower Fraser River and the Late Shuswap spawning grounds were additionally analyzed to identify microbes detected in freshwater only. Microparasite monitoring was conducted over 44 salmon microparasites, and transcriptional variation in 58 host genes involved in stress, immunity, and associated with the MRS (described by Miller et al. 2011) was assessed (Table S3).

Genetic population identification determined the lake systems to which salmon were migrating. The study was performed on two populations, Chilko and Late Shuswap, with 57 and 125 fish tagged and tracked for each, respectively (see Table S2 for experimental design). The two populations chosen have similar migration distances to reach spawning grounds (629 km for Chilko, 484 km for Late Shuswap from the mouth of the river), and in recent years, have been migrating into the river during peak river temperatures in August. This timing is normal for Chilko (a summer-run population), which has been shown to be highly resistant to stress associated with HWTs (Eliason et al. 2011; Jeffries et al. 2012). The timing is about 6 weeks early compared with historic norms for Late Shuswap (a fall-run population), which is highly susceptible to stress and mortality associated with HWTs (Jeffries et al. 2012). To minimize artefacts associated with tagging-related mortality (see Crossin et al. 2009 for details), we limited our analyses of acoustically tagged fish to those that were picked up at the first ocean receiver approximately 2 days travel time from the tagging location. The same could not be done for radiotracked fish, as radiotags cannot transmit in saltwater. Specific details on migration speeds, behavior, and mortality will not be presented herein.

Survival was assessed using days tracked and whether or not salmon arrived to spawning grounds. A PCA analysis was performed (as in Miller et al. 2011) to identify the major trajectories of the microbe data. A Pearson correlation was performed between days tracked and each of the principal components (PCs) to explore potential associations with survival. Those that were significant were used in survivorship analysis performed as outlined in Miller et al. (2011).

**Figure 3:**
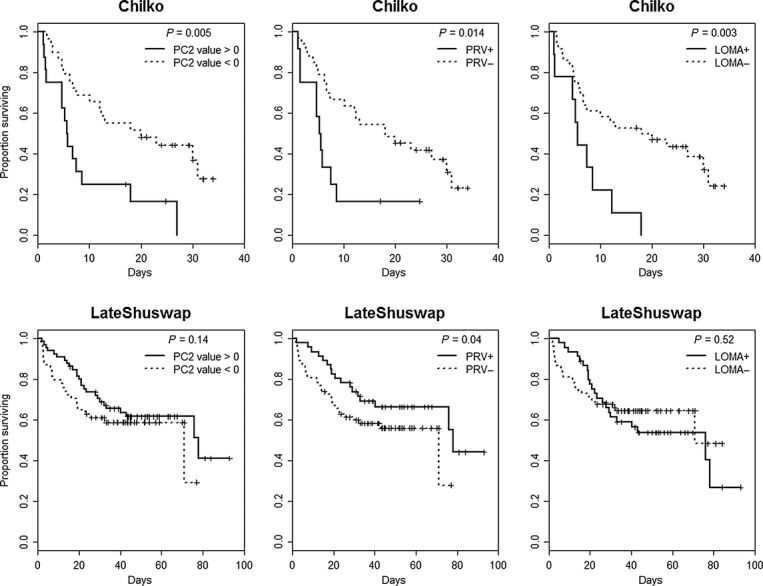
Survivorship analysis for Chilko (top) and Late Shuswap (bottom) stocks based on individual rankings for Principal Component 2 (PC2), and positive (CT<27)/negatives for Piscine Reovirus (PRV) and *Loma salmonae* (Loma) (case study II). *P*-values are shown on top right.

Seven of the 44 microparasites assessed were detectable in at least 2% of the fish tagged in the marine environment. The most prevalent microparasites were *L. salmonae* (31%), PRV (29%), *N*. *salmonis* (32%), and *F. psychrophilum* (21%). PC1 and PC2 together explained 96.9% of the total microbe variation and both were correlated with survival (*P* < 0.05). PC1 differentiated fish by the diversity of microparasites they carried, the extreme negative end comprised largely of survivors that were microbe free, and the extreme positive end containing fish carrying up to five microbes. For PC2, the positive end, which carried a disproportionate number of unsuccessful fish, was heavily loaded with *L. salmonae* and PRV, while the negative end contained more *P. theridion* positive fish. Survivorship analysis was performed separately for Chilko and Late Shuswap populations and was significant for PC2 in Chilko, for which there was a 20% differential in survival to spawning grounds (Fig. 3). Survivorship analysis was additionally performed based on *L. salmonae* and PRV positives and negatives, with both microparasites significantly associated with migration losses for the Chilko population only (Fig. 3; *P*-values cited in figures). The strongest effect on survivorship was for *L. salmonae*, whereby fish positive in the marine environment carried a 9.6 times greater odds of dying before reaching spawning grounds (*P* < 0.05); the odds ratio for PRV was 2.3 but was not significant (*P* > 0.05).

The two microparasites associated with migration survival also elicited the strongest transcriptional response in the host. Twenty of the 58 host genes were differentially expressed (after multiple test correction) in association with PRV infection, and four for *L. salmonae* (*P* < 0.0001) (Fig. S1). For PRV, genes involved in immune regulation—including complement formation (C7 and C3), T-cell activation, signaling and cytolysis (ZAP7, CD4, PRF1), interferon response (IRF1), pro-viral activity (HTATIP, EEF1AO, HNR1), viral pathogenesis (MMP25), and B-cell activation (SAMSN), and genes associated with osmoregulation (Na+K+-ATPase isoforms A1b and B1), osmotic stress (SHOP21), inflammatory response (RPL6), and feeding (TMEM18) were differentially stimulated (Fig. S1). For *L. salmonae*, ZAP7, HTATIP, EEF1AO and one unknown (C486176) associated with the MRS (Miller et al. 2011) were similarly affected. PRV is associated with an emerging disease in Europe (HSMI; Palacios et al. 2010); this is the first study documenting this virus in Sockeye Salmon and indicating any associations between PRV and disease response and/or mortality in Pacific salmon. Microsporidian salmon gill disease caused by *L. salmonae* can cause up to 30% mortality in farmed salmon (Kent and Speare 2005) and is associated with osmoregulatory dysfunction and disease in freshwater adult salmon (Table 1).

**Figure 4:**
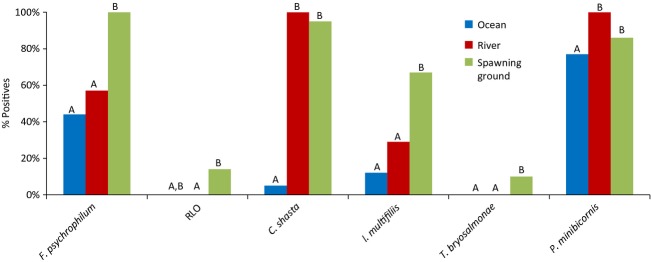
Distribution of key microbes among stocks and environments in gill tissue of return-migrating Sockeye Salmon (case study II), including bacteria *Flavobacterium psychrophilum*, Rickettsia-like organism (RLO), Myxozoa *Ceratomyxa shasta*, and *Parvicapsula minibicornis*, ciliate *Ichthyophthirius multifiliis,* and *Tetracapsuloides bryosalmonae*.

When marine and freshwater samples were analyzed together, 13 of the 44 microbes were detected, with a strong influence of environment (*P* < 1 × 10^−5^) on microbe communities. One microbe (*P. minibicornis*) increased in prevalence from the marine environment, with a slight decrease upon arrival at the spawning grounds (Fig. 4). Four microbes [*I. multifiliis*, *C. shasta, F. psychrophilum*, and Rickettsia-like organism (RLO)] were largely picked up in freshwater and increased to spawning grounds (Fig. 4) and were highly correlated with each other (*P* < 1 × 10^−6^). Coincident with increased prevalence was a significant increase in microbe diversity from the marine to freshwater environment (*P* < 1 × 10^−8^). In freshwater, the four microbes picked up in freshwater were associated with the strongest host response, with 7 to 12 of the 58 host genes affected. *I. multifiliis* and *P. minibicornis* have been associated with prespawning mortality of Sockeye Salmon in previous studies (Table 1). RLO, predominantly observed at spawning grounds, is suspected to cause skin diseases red mark syndrome or strawberry disease in Rainbow Trout in the United Kingdom and USA; this disease is not known to cause mortality but decreases the commercial value of fish (Metselaar et al. 2010). There are no studies of this organism in Sockeye Salmon.

#### Case study III

##### What microbes undergo enhanced replication in elevated river temperatures and potentially impact survival of adult salmon during river spawning migration?

Pacific salmon are ectotherms highly susceptible to changes in environmental temperature and individual susceptibility varies depending on species- and stock-specific thermal tolerance (Pörtner and Knust 2007; Farrell et al. 2008; Kent 2011; Eliason et al. 2013a). Successful completion of this once in a lifetime migration and spawning is imperative for continued propagation of Pacific salmonids and continued loss of spawners has drastic implications for future productivity. To increase our understanding of the disease potential of returning adult salmon during freshwater residence, we used a microparasite load assessment paired with measures of physiological impairment and mortality in an experimental setting. Adult Coho Salmon were collected from a tributary of the Fraser River at the Chilliwack River supplemental hatchery. Fish in ‘silver’ condition (i.e., fresh to the river) were transported to 8000-L experimental tanks at the DFO Cultus Lake Salmon Research Laboratory, Cultus Lake, BC and a subset of these fish (*n* = 9) were destructively sampled to assess microbe load upon collection. At arrival, tank temperatures were equal to that of the river (10°C); after a 24-h recovery period, the temperature in half of the tanks was increased to 15°C over 48 h, yielding two temperature treatments designated ‘cool’ and ‘warm’, with two tank replicates per temperature. After experimental day 14, a subset of survivors from each temperature group was destructively sampled (cool: *n* = 17; warm: *n* = 18); the remaining fish were sampled at experimental day 24 (cool: *n* = 10; warm: *n* = 7). Tissue samples from gill, liver, spleen, kidney, heart, muscle, and brain were homogenized separately, then 20 *μ*L of aqueous phase from each tissue was pooled to capture the maximum breadth of microbe diversity within each individual. Molecular analysis was carried out following protocols described previously, and the experimental design is presented in Table S2.

To avoid any unknown effect of tissue degradation on microbe load, only surviving fish were used in this analysis. Total mortality was far greater among warm fish than cool (39% and 3%, respectively; Chi squared test: *P* < 0.001). Given evidence of enhanced mortality of adult salmon at HWT (e.g. Jeffries et al. 2014; this study), our hypotheses were constructed within a framework of a microbe load threshold. In brief, we predicted microbe loads to increase across all individuals with time due to senescence and other natural factors contributing to decreased resistance of the host (see discussion above). Although microparasite replication may be restricted, senescing fish would not be expected to reduce microbe loads within their tissues (A. K. Teffer, unpublished data). Hence, a threshold load would ultimately be reached and either maintained (survivors) or advance to result in host morbidity. High-load fish would thus drop out of the population and average loads of surviving fish would remain unchanged over time. However, if HWT diminishes the resiliency of the host to infection, the load threshold for survival would be decreased at HWT, especially later in the migration and further along senescence trajectory. Therefore, we hypothesized that while cool survivors would show no difference in microparasite loads between 14 and 24 days, warm survivors would show a decrease in average loads and truncation of load distributions at 24 days relative to 14 days, evident of decreased threshold load levels and a loss of high-load individuals at HWT. Furthermore, this effect is only expected to be evident for pathogenic microbes (e.g., *P*. *minibicornis*), as nonpathogenic microparasites (e.g., *K. thyrsites*) could theoretically be present in high loads without causing mortality (Table 1).

**Figure 5:**
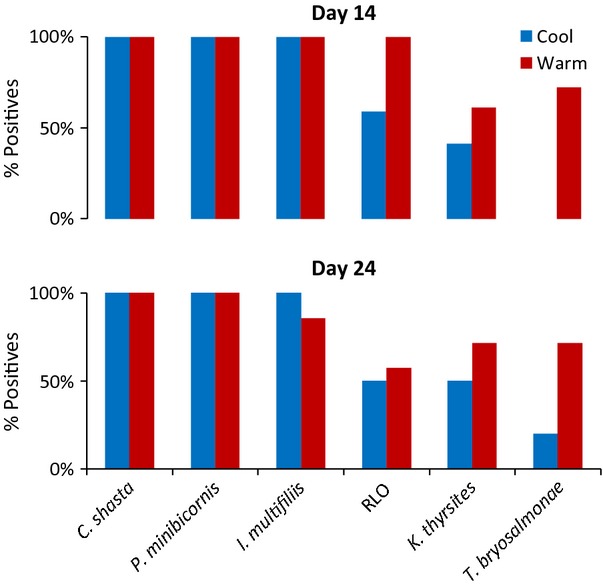
Percent prevalence of detections (CT <27) of four myxozoan parasites (*Ceratomyxa shasta*, *Parvicapsula minibicornis*, *Kudoa thyrsites*, and *Tetracapsuloides bryosalmonae*), one ciliate (*Ichthyophthirius multifiliis*) and one bacteria (Rickettsia-like [RLO]) present at experimental days 14 and 24 in case study III.

**Figure 6:**
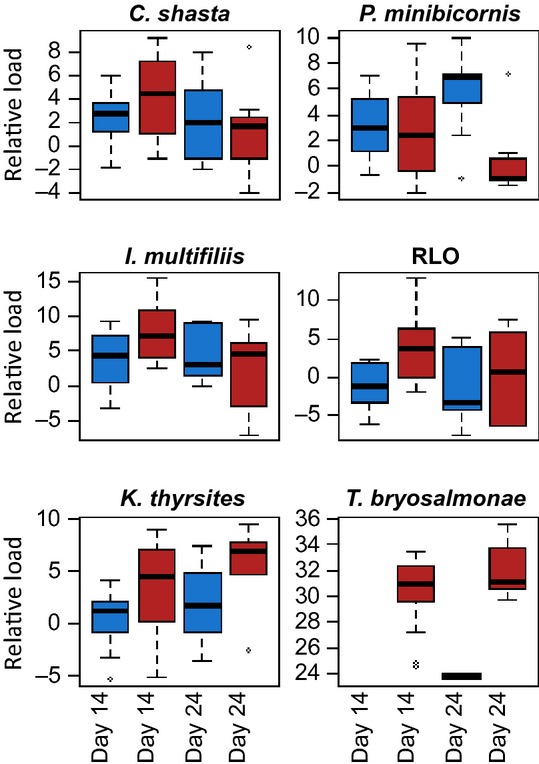
Box plots contrasting the distributions of relative loads (50 – CT) of four myxozoan parasites (*Ceratomyxa shasta, Parvicapsula minibicornis, Kudoa thyrsites* and *Tetracapsuloides bryosalmonae*), one ciliate (*Ichthyophthirius multifiliis*) and one bacteria [Rickettsia-like organism (RLO)] of adult Coho Salmon at collection (Day 1; *n* = 9), after 14 days held at either cool (10°C; *n* = 17) or warm (15°C; *n* = 18), and after 24 days (cool: *n* = 10; warm: *n* = 7) (case study III).

Four myxozoan parasites (*C. shasta*, *P. minibicornis*, *K. thyrsites,* and *Tetracapsula bryosalmonae*), one ciliate (*I. multifiliis*) and one bacteria (Rickettsia-like organism, RLO) were >50% prevalent in collected fish and incorporated into multivariate testing. Prevalence was largely unchanged in both cool and warm treatments (Fig. 5). However, results show significant differences in microbe loads between temperature groups (Fig. 6; manova: *P* = 0.001) and a significant interaction between temperature and time (*P* = 0.045). This interaction was primarily attributable to temporal differences at HWT, though not significant after Bonferroni adjustment (*P* > 0.0001). Marginal differences in the relative loads of *C. shasta*, *I. multifiliis*, RLO and *P. minibicornis*, though not statistically significant (*P* > 0.0001), were apparent between 14 days and 24 days at HWT, each consistent with losses of high-load individuals. There was no similar trend among cool temperature fish, and in fact, *P. minibicornis* loads were marginally higher, but not significantly so, at 24 days than 14 days. Our low sample sizes likely attributed to our inability to identify statistically significant differences, but trends in the data warrant further examination of how microbe load and composition respond to HWT stress within a survival context. We identify *C. shasta*, *I. multifiliis,* and RLO as potentially pathogenic organisms affecting adult coho salmon survival and support nonpathogenic designation despite enhanced replication at HWT of *K. thyrsites* and *T. bryosalmonae*. *Ichthyophthirius multifiliis* is known to have a thermally responsive life cycle (Ewing et al. 1986; Noe and Dickerson 1995; Matthews 2005; Aihua and Buchmann 2001) and has been identified as pathogenic in BC previously in the Skeena River, associated with high levels of prespawn mortality of returning adult sockeye salmon (Traxler et al. 1998). Detrimental effects of *P. minibicornis* infection on the health and survival of adult salmon in the Fraser River have been demonstrated (Wagner et al. 2005; Crossin et al. 2008. Bradford et al. 2010) and our data complement previous findings by showing how cool temperature may enhance the ability of fish to maintain high microparasite loads. Clearly, interactions between microparasites within a temperature context as well as interactions with other stressors (e.g., fisheries capture) warrant further evaluation. Such an investigation is currently underway using repeated nonlethal sampling techniques to measure microbe load, reproductive status and physiological impairment of individuals throughout freshwater residence (A. K. Teffer, unpublished data).

#### Case study IV

##### Do salmon that fall prey to predators have higher microparasite diversity or load than those in the general population?

Studies on Red Grouse (*Lagopus lagopus scotica*) and *Daphnia* support the hypothesis that infected animals are more prone to succumb to predation (Hudson et al. 1992; Johnson et al. 2006). To determine whether similar patterns exist in the ocean, we used predation by a colonial seabird as a model system to test the null hypothesis that the prevalence and/or load of microparasites in post-smolts migrating in the ocean do not impact their susceptibility to predators. Additional factors associated with salmon condition, environmental factors (oceanographic conditions and prey quality), and genetic (population) factors that primarily influence body condition and health of salmon smolts will be pursued in subsequent studies.

In the eastern North Pacific, the timing of the seaward migration of Pink, Chum, and Sockeye Salmon smolts coincides with the chick-provisioning period of Rhinoceros Auklets. Taking advantage of the fact that birds deliver whole fish to nestlings, we sampled auklet diets intensively at several large breeding colonies in BC. Sampling on colonies coincided with coast-wide trawl surveys, enabling us to directly compare characteristics of auklet-predated smolts against control, trawl-caught smolts. Using this approach, we have established very clearly that the auklets disproportionately take smolts that are small and in poor condition (S. Tucker, unpublished data); herein, we examine the microparasite profiles of Sockeye Salmon to determine whether or not those that are predated have higher incidence and/or load of microparasites than those in the general marine environment where the auklets are feeding. We additionally assessed whether microbe profiles were influenced by size and condition (measured as the residuals of the length-weight relationship over all fish sampled, and categorizing fish as above or below average mass to body length). Our null hypothesis is that microbes will not be associated with variance in size or condition factor.

Eighty-six Sockeye Salmon post-smolts collected from a trawl survey within Queen Charlotte Sound, BC were measured for fork length and weight. Seventy-nine Sockeye Salmon post-smolts were collected from auklet nesting colonies in Queen Charlotte Sound and treated similarly. Gill and liver tissues were combined for the monitoring of 40 microbes.

**Figure 7:**
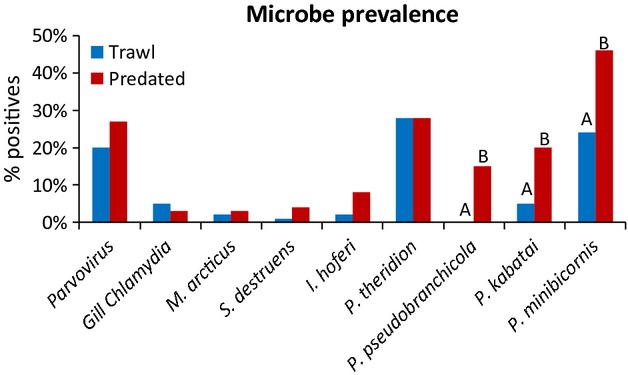
Percent prevalence of microbes (CT <27) identified in Sockeye Salmon from samples predated upon by Rhinoceros Auklet compared to those from a trawl survey in waters adjacent to sampled colonies (case study IV). A/B indicates a significant difference between groups, tested by microbe.

Thirteen of the 40 microparasites surveyed amplified products with CT<27 (Table 2; Fig. 7). manova was applied to determine the relative roles of predation, size and condition factor, and their interaction terms, in the variances in microbe distributions among the 13 detected microparasites.

**Figure 8:**
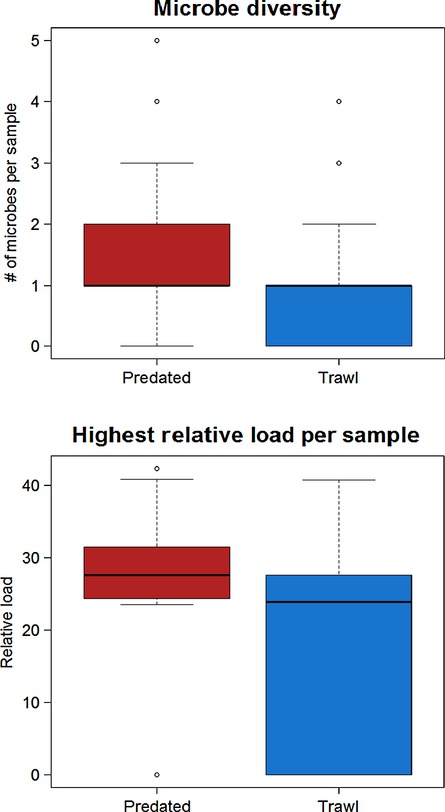
Box plots revealing differences identified in microbes found in Sockeye Salmon from samples predated upon by Rhinoceros Auklet compared with those from a trawl survey in waters adjacent to sampled colonies (case study IV). (A) Microbe diversity, indicating the number of distinct microbes carried per sample. (B) Highest relative load (50-CT) of any microbe within samples.

Microparasite distributions were differentiated between predated and trawl samples (*P* < 0.001), but no significant relationship with size, condition factor, or any interactive terms was observed (*P* > 0.05). Post hoc testing revealed significantly higher levels of three *Parvicapsula* parasites—*P*. *pseudobranchicola* (*P* < 0.001), *P. kabata* (*P* < 0.005), and *P. minibicornis* (*P* < 0.005)—in the predated Sockeye. *P. minibicornis* was highly prevalent with detection in 46% of predated fish and 24% in the general population (trawl) and a minimum CT of 7.7, indicative of a load of >10^7^ (Fig. 7). *P. kabatai* was observed in 20% of predated fish and 4% of trawled, with a minimum CT of 15.3—load >10^3^. *P. pseudobranchicola* was observed in 16% of predated and no trawled fish, with a minimum CT of 19.1—load >10^2^ Predated fish also carried a higher diversity (*P* < 0.001) and load (*P* < 0.001) of microparasites than those in the general population (Fig. 8). Individual fish carried between 0 and 5 microparasites, with an average of 1.6 for predated and 0.9 for the general population. The vast majority of fish with >3 microparasites were predated, as were 11 of the 14 fish with 3 microparasites. Thirty-nine percent of predated fish carried at least one microbe with a CT<20 (load > 10^4^) versus only 16% in the general population. Moreover, whereas 6% of predated fish carried two microparasites at high load, none of the general population samples carried more than one high-load microparasite.

These data refute the null hypothesis that the condition of fish, as defined by the microparasites they carry, does not affect their probability of predation. While the majority (87%) of Sockeye Salmon post-smolts that were preyed upon by auklets were in poor body condition (S. Tucker, unpublished data), there was no relationship between condition and the microparasites they carry. However, how the presence of these parasites might contribute to the overall physical condition of fish, or what the manifestations might be with respect to health or to factors affecting predator avoidance remains unknown. Of the three myxozoan *Parvicapsula* parasites showing a significant association with predation, only *P. minibicornis* has been extensively studied in wild salmon, and then only in adults. *P*. *minibicornis* is picked up in freshwater where its’ alternate host, the polychaete *Manayunkia speciosa* (Bartholomew et al. 2006), resides. In return-migrating salmon, *P. minibicornis* is associated with severe infection and pre-spawning mortality after 450°C accumulated thermal units in the river (Wagner et al. 2005; Bradford et al. 2010); similar studies in smolts have not been published. *P. pseudobranchicola* is a marine parasite that has been associated with mortalities of farmed Atlantic Salmon in Northern Norway (Table 1; Karlsbakk et al. 2002), with impacts on swimming activity level and possibly vision (bleeding around the eyes) (Jørgensen et al. 2011), which could affect predator avoidance. *P. pseudobranchicola* infection levels in Norway are higher in farmed than wild fish (Jørgensen et al. 2011). *P. kabatai* was first isolated from kidneys of Pink Salmon in Quinsam River, BC (Jones et al. 2006), and histologic lesions associated with the parasite from wild-collected fish have been described (Saksida et al. 2012). Dual infections with myxozoan parasites can be a common occurrence and may have a synergistic effect increasing lethality of infection (Nichols and True 2007).

## Discussion

Disease consequences not only depend on the spatial and temporal scale of a pathogen but also its impact in terms of mortality and morbidity (Peeler et al. 2007). Most studies of infectious agents impacting wild populations are association-based—that is, they document parasite distributions (e.g., Margolis and Arthur 1979; McDonald and Margolis 1995; Bennett et al. 1998; Kent et al. 1998; Arkoosh et al. 2004; Sandell 2010; Ferguson et al. 2012), and occasionally assess shifts in prevalence and/or load to develop hypotheses about impacts (e.g., Gordon and Rau 1982; Kalbe et al. 2002; Jacobson et al. 2008), but do not measure mortality directly. While our case studies also take an association-based approach, the study merging acoustic tracking with microparasite monitoring was able to directly associate specific microbes with migration success, resolving two infectious agents, a microsporidian parasite (*L. salmonae*) and a virus (PRV), that were correlated with premature migration mortality in one of two assessed stocks, and especially notable during marine migration. Moreover, we showed that the microparasites most predictive of fate stimulated the strongest immune response in the host. In the predation study, we directly compared microparasite profiles of salmon being predated by auklets with those in the general population and showed that post-smolts carrying any of three species of myxozoan *Parvicapsula* parasites were more likely to be predated. Moreover, we found that predated fish generally carried higher microparasite diversity and load. Climate shifts are expected to continue to impose further stress on already declining populations of salmon, creating an optimal environment for a range of infectious diseases to flourish. In a holding study, we showed that myxosporean parasites (*P. minibicornis* and *C. shasta*) and the etiological agent associated with strawberry disease (RLO) were responsive to temperature shifts in freshwater, rapidly increasing in load in Coho Salmon held at 10°C and 15°C, and then showing a truncated load distribution among 15°C survivors, suggesting a loss of high-load individuals at high temperature. These data corroborate those of previous studies indicating temperature-mediated responses of *C. shasta* and *P. minibicornis* infecting Chinook and Sockeye Salmon, respectively; salmon are exposed to both of these parasites in freshwater and both negatively impact survival (Table 1). As a whole, these studies highlight the potential importance of myxozoan and microsporidian parasites in wild salmon.

Microsporidia are related to fungi and infect a broad spectrum of taxa, with half of the known genera infecting aquatic hosts (Stentiford et al. 2013). In aquatic systems, their impacts range from cryptic to catastrophic, with the potential to drive host population cycles and ecological impact on species interactions within ecosystems (Stentiford et al. 2013). Microsporidian parasites have also been implicated in colony collapse disorder in bees (Higes et al. 2008) and are the most common infections among immuno-compromised humans (Williams 2009). Myxozoans are highly diverse spore producing parasites that share a close phylogenetic relationship with cnidarians (Chang 2013). Myxozoans are largely aquatic, most with obligate development in teleost fish and annelid worm hosts (Kent et al. 2001). While their complex life cycle may reduce the likelihood of their sustaining persistent disease epidemics in the wild (Bakke and Harris 1998), three have been implicated in disease outbreaks in wild and cultured salmon (*M. cerebralis* causing whirling disease, *T. bryosalmonae* causing PKD, and *C. shasta* causing ceratomyxosis) and one has caused severe economic impacts on industry (*K. thyrsites* causes post-harvest myoliquefaction of muscle tissue; Kent et al. 2001).

Coevolution between microparasites and their hosts will be most strongly felt in systems where population-level impacts of pathogens occur. However, population-level effects may be reduced in systems with strong density dependence, especially if infection-related mortalities occur before density dependence is strongest (Fujiwara et al. 2014). In salmon, infection-related mortality within populations that occurs just prior to smoltification may be less affected by density dependence, as competition for resources in the marine environment would not be limited to the size of one or a few populations, but the combined densities of hundreds of merging populations. However, if large-scale mortalities were to occur during early marine residence, it is possible that reduced competition for resources could counter the negative impacts of infectious disease. However, on the west coast of Canada, competition for resources is hypothesized to be largely driven by the massive explosion of even year Pink Salmon populations (Ruggerone et al. 2003); hence, unless Pink Salmon were to also be affected, densities may not be reduced to sufficiently low levels to counter the impacts of disease. In the case of return-migrating salmon, disease-associated prespawn mortality may have a lower impact on population variance in years with high returns as density-dependent competition for spawning resources may counter impacts of disease. However, in years with low-density returns and high river temperatures excelerating the rate of disease development and reservoirs of microparasites with alternate hosts, strong population-level effects of disease may be felt. Interestingly, in Sockeye Salmon, abundance of returns has been relatively stable in the dominant cycle year where millions of salmon return to spawn relative to other years that have experienced strongly declining abundance (Peterman and Dorner 2011), consistent with a relatively stronger impact of disease on populations when densities are low. The hypothesis that density dependence may reduce the impact of infectious disease at a population-level is contrary to microbe-host evolutionary theory, hence requires further study.

Multiple infections by various microparasites of salmonids present further complexity to the host/parasite/environment relationship (Thomas and Blanford 2003). The microbe-monitoring data from all four case studies revealed a high percentage of BC salmon carrying coinfections of multiple microparasites. However, it was relatively rare for fish to carry high loads of multiple microbes at once (maximum observed in our studies was three microbes at CT<20 carried in a single tissue sampled from live fish). Given the range of infectious agents salmon are exposed to in their life time, and the potential that many microparasites may go into a carrier state upon recovery from disease (Table 1), coinfections are expected. The question is, are fish that carry higher microbe diversities in a generally poorer conditional state than those with lower diversity? The answer to this question probably depends upon the composition of the coinfection. In the predation study, we showed a direct correlation between microbe diversity and predation, suggesting a poorer conditional state associated with microbe diversity. Similarly, Jacobson et al. (2008) and Sandell (2010) found that in surveys of post-smolts in the ocean, higher parasite diversity, prevalence and loads within fish were observed in years of good relative to poor ocean productivity, and concluded that parasite infections were less tolerated when fish were otherwise stressed. There is also evidence from laboratory studies that particular coinfections may impact the pathogenicity of single microbes, essentially affecting their clinical and immunological evolution. For example, some viruses cannot replicate efficiently without coinfection; many parvoviruses in the dependovirus genus require coinfection with adenoviruses for replication (Anderson and Pattison 1984). A salmon parvovirus that was recently identified by NGS in Sockeye Salmon is phylogenetically close to the dependovirus genus, but whether it requires coinfection for replication is not yet known (KM Miller, unpublished data). Diseases stimulated by coinfections between viruses and microsporidian parasites are also common (Duncan et al. 2012; Toplak et al. 2013), but have not been studied extensively in fish. Given the very large number of microsporidian parasites observed in fish (Stentiford et al. 2013), this is an important area of future study. Alternately, some coinfections may be beneficial to the host. For example, in cell cultures, IPNV interferes with the IHNV replication (Alonso et al. 2003) and induces interferon activity which may act to suppress IHNV replication (de Kinkelin et al. 1992). As well, in trout infection with avirulent cutthroat reovirus induces an IHNV-resistant state (Hedrick et al. 1994).

Predators may limit levels of infection within populations, thereby decreasing rates of host-pathogen coevolution. In a culture setting, rapid removal of sick or dead fish is an effective way to keep disease under control (Jarp and Karlsen 1997; Murray and Peeler 2005). In natural environments, predation, natural and anthropomorphic (fishing) may reduce infectious disease by reducing host densities below certain thresholds (Dobson and May 1987). For microparasites primarily exchanged horizontally, if predators select infected fish at early stages of disease, they could decrease the threshold of infection-related mortality, thereby decreasing exchange rates of microparasites and reducing the probability of epidemic levels of infection (Lafferty and Gerber 2002). In doing so, natural predation may increase the costs of high pathogen virulence if moribund fish are removed before transmission occurs, which would, in essence, decrease the rate of coevolution among microparasites and their hosts. In fisheries, if certain gear types were shown to selectively harvest fish infected with important disease-causing pathogens, under some circumstances, evolutionary disease management strategies may actually warrant harvesting a portion of affected stocks to minimize disease impacts at spawning grounds. Alternately, for microparasites with alternate hosts, like the myxozoans in our predation study, predators could simply increase the probability of infection-related mortality, thereby increasing the potential for coevolution, although this effect could be countered by reduced infection levels in the alternate host.

The ecological and evolutionary outcomes of cumulative impacts of climate, infection, and predation are hard to predict, as their direction will depend upon predator and host densities and how strongly temperature impacts microparasite replication rates and swim performance. Temperature can immunocompromise the host and increase the replication rate of numerous microparasites, increasing rates of infection and disease development; case study III corroborates this assertion. The cumulative impact of temperature and infection in the absence of predation should therefore increase rates of coevolution, especially in susceptible hosts. In a system with predators, impacts on swim performance may be felt at a lower level of infection when temperatures are elevated, increasing the vulnerability of fish to predation. If the density of predators is sufficiently high to reduce salmon densities while disease levels are still low, microparasite evolution may be reduced. However, if temperature impacts on microparasite replication rates are faster than predators can remove impacted fish, predators would have limited impact. In essence, the arms race of host-microparasite evolution in wild populations could be enhanced with environmental stress and decreased under high levels of predation.

There is not likely a single stressor that can account for the massive declines in productivity and abundance of salmon in the northeast Pacific; rather the cumulative and interactive effects of multiple stressors are likely at play. The uncertainties in predicting evolutionary responses to cumulative stressors are considerably greater for organisms such as salmon that have complex, migratory life cycles (Crozier et al. 2008), as responses at one stage of development may impact subsequent states, and resistance may not impact all life-history stages equally. It is imperative that we build a greater understanding of both plastic and evolutionary responses to individual stressors and determine whether cumulative effects are additive, antagonistic, or synergistic if we are to predict the outcomes of cumulative stressors and variation in population-level responses.

Moving forward, there are many ways that modern technologies can improve the depth and breadth of ecological and evolutionary information required to assess the impacts of disease processes in natural systems. Merging of broad-scale microbe surveillance with biotelemetry and assessments of cumulative stressors will provide greater insight into the microbes of most import to wild populations. Indeed, such multidisciplinary approaches are demanded by complex environmental problems (Cooke et al. 2008). Evolutionary drivers of variation in microparasite susceptibility can additionally be incorporated into these ‘natural’ studies by linking data on MHC variation or by taking a dQTL approach. Gene expression profiling through microarrays or high-throughput biomarker surveillance of host immune genes can be integrated to elucidate the microparasites that elicit strong responses in the host and for which MHC-related defence mechanisms are important. These indirect correlative approaches on naturally migrating wild organisms will allow for the ‘discovery’ of potential linkages between microparasites, genetic susceptibilities, and probability of disease (via levels of immune stimulation) that can be followed up in laboratory studies to better understand mechanistic linkages with disease.

## Supporting Information

**Data S1.** Fluidigm BioMark Methods. **Table S1.** Overview of the microparasites for which genetic associations with disease resistance, identification of disease-related quantitative trait loci (dQTL), and/or host response through microarray gene expression profiling studies have been determined. **Table S2.** Design of case studies I (A), II (B and C), and III (D). **Table S3.** Host gene Taqman assays assessed in Case Study II. **Figure S1.** Gene expression of the 20 host genes significantly associated with PRV infection in case study II.
