# Supplementary material for: Infectious disease, shifting climates, and opportunistic predators: cumulative factors potentially impacting wild salmon declines
Source: Evol Appl. 2014 May 27;7(7):812–55. doi: 10.1111/eva.12164 (PMC4227861; doi:10.1111/eva.12164)
Supplement: Supplementary file 1 — Data S1. Fluidigm BioMark Methods. Table S1. Overview of the microparasites for which genetic associations with disease resistance, identification of disease-related quantitative trait loci (dQTL), and/or host response through microarray gene expression profiling studies have been determined. Table S2. Design of case studies I (A), II (B and C), and III (D). Table S3. Host gene Taqman assays assessed in Case Study II. Figure S1. Gene expression of the 20 host genes significantly associated with PRV infection in case study II. [file eva0007-0812-sd1.docx]

Data S1

Fluidigm BioMark Methods

With microfluidic PCR, the sample volumes are 1/1,000^th^ that of normal PCR (7 nl compared to 7-12 μl), hence a specific target amplification (STA) enrichment is performed prior to the microfluidic qPCR. The STA reaction is a multiplex PCR, which uses the primers (no probes) used for the qRT-PCR at 1/20 of concentration to run 14 PCR cycles. STA reactions are diluted 1:5 and primers removed prior to microfluidics PCR, which runs each assay separately. Five microlitre aliquots of sample premix (1x Taqman PCR master mix (Lifetech), 1x GE sample loading reagent (Fluidigm), and 2.25 μl 5-fold diluted STA product) and 5 μl of the assay premix (1x assay loading reagent (Fluidigm), 10 μM each primers, and 3 μM probe) were loaded into the 96.96 dynamic array (Fluidigm) and mixed with an IFC controller HX (Fluidigm). PCR was performed under the following conditions: 50^o^C for 2-min, 95^o^C for 10-min, followed by 40 cycles of 95^o^C for 15-s, 60^o^C for 1-min. The quantification cycle (Cq, also known as the threshold cycle, CT) was determined using Fluidigm Real-Time PCR Analysis software 3.0.2 (Fluidigm). For each run, 2-3 housekeeping gene controls were used to assess the cDNA quality, and up to 5 negative processing controls (extraction, cDNA synthesis) were included. We also included in each run 5 serial dilutions of artificial positive control (APC) clone standards for each microparasite assay (APC’s combined into a single sample) to assess assay performance and calculate copy number. These controls contain a generic sequence that can be additionally probed to identify positive clone standard contamination (described in Snow *et al.* 2009). The microparasite assay probes were labeled with 6-FAM, while the APC probes were labeled with NED. If any sample had APC (NED) positives with any microbe, it was removed from analysis.

Table S1. Overview of the microparasites for which genetic associations with disease resistance, identification of disease-related quantitative trait loci (dQTL), and/or host response through microarray gene expression profiling studies have been determined.

| **Microbe** | **Agent** | **Genetic Associations** | **dQTL** | **Microarray Studies** |
| --- | --- | --- | --- | --- |
| *Aeromonas salmonicida* | Bacteria | Gjedrem *et al.* 1991 |  | Ewart *et al.* 2005, 2008 |
| *Flavobacterium psychrophilum* | Bacteria | Henryon *et al.* 2005; Leeds *et al*. 2010 |  |  |
| *Piscirickettsia salmonis* | Bacteria |  |  | Rise *et al.* 2004 |
| *Renibacterium salmoninarum* | Bacteria | Withler and Evelyn 1990; Beacham and Evelyn 1992 |  |  |
| Rickettsia-Like Organism | Bacteria | Lloyd *et al.* 2011 (trout) |  |  |
| *Vibrio anguillarum* | Bacteria | Beacham and Evelyn 1992; Gjøen *et al.* 1997 |  | Ching *et al*. 2010 |
| *Vibrio salmonicida* | Bacteria | Gjedrem and Gjoen 1995 |  |  |
| *Yersinia ruckeri* | Bacteria | Henryon *et al.* 2005 |  | Harun *et al.* 2011 |
| Fish Viruses | Virus |  |  | Verrier *et al*. 2011; Novoa *et al.* 2010; Krasnov *et al.* 2011 |
| Infectious haematopoietic necrosis virus | Virus | Arkush *et al.* 2002; Amend and Nelson 1977 | Verrier *et al.* 2013b | Purcell *et al.* 2006; Miller *et al.* 2007 |
| Infectious pancreatic necrosis virus | Virus | Storset *et al.* 2007 | Ozaki *et al.* 2001; Houston *et al.* 2008a, 2008b, 2010 | Marjara *et al.* 2011; Krasnov *et al.* 2011 |
| Infectious salmon anemia virus | Virus | Kjøglum *et al.* 2008 | Moen *et al.* 2004, 2007 | Workenhe *et al.* 2009; Krasnov *et al.* 2011; Jorgensen *et al.* 2008 |
| Pacific salmon parvovirus | Virus |  |  |  |
| Piscine myocarditis Virus | Virus |  |  | Gerrit *et al.* 2011; Timmerhaus *et al.* 2011; Krasnov *et al.* 2011 |
| Piscine reovirus | Virus |  |  | Krasnov *et al.* 2011 |
| Salmon alphavirus 1, 2, and 3 (PD/SD/HSS) | Virus | Norris *et al.* 2008 |  | Herath *et al.* 2012; Larsson *et al.* 2012 |
| Viral hemorrhagic septicemia virus | Virus | Dorson *et al.* 1995 | Verrier *et al.* 2013a |  |
| *Gyrodactylus salaris* | Ectoparasitic worm | Bakke *et al.* 1990 |  |  |
| *Neoparamoeba perurans* | Amoeba |  |  | Morrison *et al.* 2006 |
| *Myxobolus cerebralis* | Myxozoan | Arkush *et al.* 2002 |  |  |

Table S2. Design of case studies I (A), II (B and C), and III (D).

A

Table Note: In case study I, liver tissue was destructively sampled

from return migrating sockeye salmon originating from four stocks

(Harrison, Quesnel, Chilko, and Shuswap/LateShuswap) and

sampled in three environments (Ocean, River and Spawning)

over six years from 2005-2010.

B

Table Note: In case study II, adult gill biopsies were non-destructively sampled from

acoustic and radio tracked sockeye migrating towards spawning grounds in 2010.

Migration timing and fate were determined; here we show fate classified as ocean

mortality, river mortality or survivor to spawning grounds.

C

Table Note: In case study II, adult gill tissue was destructively

sampled across three environments (Ocean, River, Spawning)

and two stocks (Chilko,Late Shuswap) in 2010 migrating fish.

D

Table Note (In case study III, adult Chilliwack River hatchery Coho

were sampled for gill, liver, spleen, kidney, heart, muscle and brain

in 2012, with all tissues homogenized for microbe analysis. Nine

initial samples were taken at the onset of holding and the remaining

samples were taken after 14 or 24 days held in cool (10 ^o^C) or warm

water (15 ^o^C).

Table S3. Host gene taqman assays assessed in case study II.

Table Note: Case study II host gene Taqman assays assessed for gene expression of 58 biomarkers and three housekeeping genes, MrpL40, Coil-P84-2, and 78d16.1. Gene and assay names with accession numbers and primer/probe sequences presented for each assay.


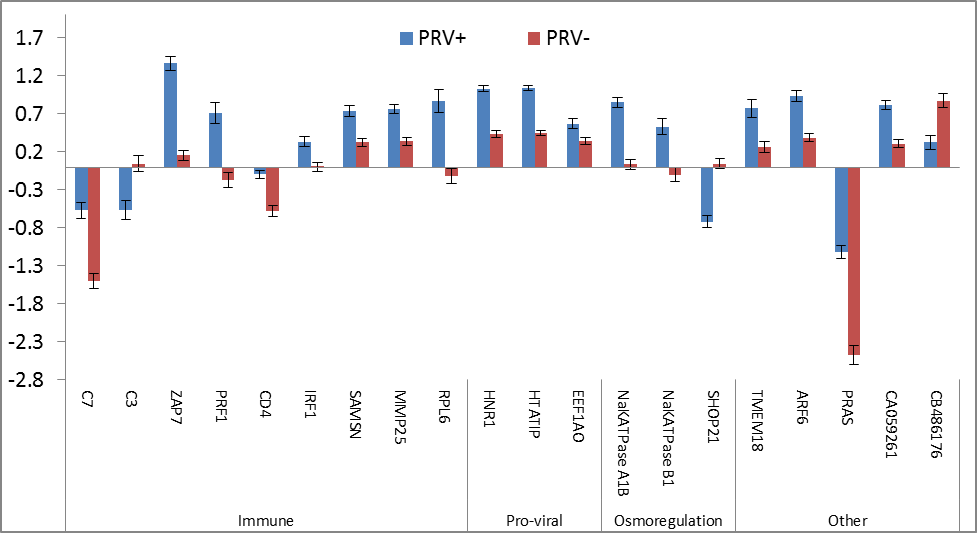


Figure S1. Gene expression of the 20 host genes significantly associated with PRV infection in case study II. Standard error noted by bars.

Literature S1 (cited in Tables)

Actis, L. A., M. E. Tolmasky, and J. H. Crosa 1999. Vibriosis. In: P. T. K. Woo, and D. W. Bruno, eds. *Fish Diseases and Disorders*, Volume 3. Viral, Bacterial and Fungal Infections. pp. 523-557. CAB International, Oxon, United Kingdom.

Amend, D. F. and J. R. Nelson 1977. Variation in the susceptibility of sockeye salmon *Oncorhynchus nerka* to infectious hematopoietic necrosis virus. *Journal of Fish Biology* **11**:567–573.

Andersen, L., A. Bratland, K. Hodneland, and A. Nylund 2007. Tissue tropism of salmonid alphaviruses (subtypes SAV1 and SAV3) in experimentally challenged Atlantic salmon (*Salmo salar* L.). *Archives of Virology* **152**:1871-1883.

Arkush, K. D., A. R. Giese, H. L. Mendonca, A. M. McBride, G. D. Marty, and P. W. Hedrick 2002. Resistance to three pathogens in the endangered winter-run chinook salmon (*Oncorhynchus* *tshawytscha*): effects of inbreeding and major histocompatibility complex genotypes. *Canadian Journal of Fisheries and Aquatic Sciences* **59**:966–975.

Austin, B. and D. A. Austin 1993. *Bacterial Fish Pathogens: Disease in Farmed and Wild Fish*, 2nd edition, Ellis Horwood Limited, Chichester, UK.

Bahar, M. W. , L. P. Sarin, S. C. Graham, J. Pang, D. H. Bamford, D. I. Stuart and J. M. Grimes 2013. Structure of a VP1-VP3 complex suggests how birnaviruses package the VP1 polymerase. *Journal of Virology* **87**:3229–3236.

Bakke, T.A., P. A. Jansen, and L. P. Hansen 1990. Differences in the host resistance of Atlantic salmon, *Salmo salar*, stocks to the monogenean *Gyrodactylus* *salaris* Malmberg, 1957. *Journal of Fish Biology* **37**:577–587.

Baldwin, T. J., E. R. Vincent, R. M. Silflow, and D. Stanek 2000. *Myxobolus cerebralis* infection in rainbow trout (*Oncorhynchus mykiss*) and brown trout (*Salmo* *trutta*) exposed under natural stream conditions. *Journal of Veterinary Diagnostic Investigation* **12**: 312-321.

Bartholomew, J. L. 2009. Long-term fish disease monitoring program in the Lower Klamath River. BOR annual report. Oregon State University annual report. <http://microbiology.science.oregonstate.edu/files/micro/images/Barthol_BOR_report_2008_OSU.pdf>

Bartholomew, J. 2010. Long-Term fish disease monitoring program in the Lower Klamath

River Annual Report for 2009. Department of Microbiology, Oregon State University, Corvallis, Oregon. (available at <http://microbiology.science.oregonstate.edu/files/micro/images/BOR_report_2009_April_2010_OSU.pdf>.

Baulaurier, J., N. Bickford, J. L. Gregg, C. A. Grady, A. Gannam, J.R. Winton and P. K. Hershberger 2012. Susceptibility of Pacific herring *Clupea pallasii* to viral hemorrhagic septicemia (VHS) is influenced by diet. *Journal of Aquatic Animal Health* **24**:43-48.

Beacham, T. D., and T. P. T. Evelyn 1992. Population and genetic variation in resistance of Chinook salmon to vibriosis, furunculosis, and bacterial kidney disease. *Journal of Aquatic Animal Health* **4**:153–167.

Bettge, K., H. Segner, R. Burki, H. Schmidt-Posthaus, and T. Wahli 2009. Proliferative kidney disease (PKD) of rainbow trout: temperature-and time-related changes of *Tetracapsuloides bryosalmonae* DNA in the kidney. *Parasitology* **136**: 615-625.

Bowser, P. R., J. W. Casey, J. R. Winton, and A. E. Goodwin 2009. Major losses of wild fish in the USA from a novel strain of viral hemorrhagic septicemia virus (VHSV). *Israeli Journal of Aquaculture* **61**:270-271.

Bradford, M. J., J. Lovy, D. A. Patterson, D. J. Speare, W. R. Bennett, A. R. Stobbart, and C. P. Tovey 2010. *Parvicapsula minibicornis* infections in gill and kidney and the premature mortality of adult Sockeye salmon (*Oncorhynchus nerka*) from Cultus Lake, British Columbia. *Canadian Journal of Fisheries and Aquatic Sciences* **67**:673-683.

Brocklebank, J., and S. Raverty 2002. Sudden mortality caused by cardiac deformities following seining of preharvest farmed Atlantic salmon (*Salmo salar*) and by cardiomyopathy of post-intraperitoneally vaccinated Atlantic salmon parr in British Columbia. *Canadian Veterinary Journal* **43**:129–130.

Bullock, G. L., and R. L. Herman 1988. Bacterial kidney disease of salmonid fishes caused by *Renibacterium salmoninarum*. Fish Disease Leaflet 78. US Department of the Interior, US Fish and Wildlife Service, Washington, D.C., 8 pp. Available at: <http://pubs.er.usgs.gov/publication/2001381>

Cavender, W. P., J. S. Wood, M. S. Powell, K. Overturf, and K. D. Cain 2004. Real-time quantitative polymerase chain reaction (QPCR) to identify *Myxobolus cerebralis* in rainbow trout *Oncorhynchus mykiss*. *Diseases of Aquatic Organisms* **60**:205–213.

Ching, B., S. Jamieson, J. W. Heath, D. D. Heath, and A. Hubberstey 2010. Transcriptional differences between triploid and diploid Chinook salmon (*Oncorhynchus tshawytscha*) during live *Vibrio anguillarum* challenge. *Heredity* **104**:224–234.

Clifton-Hadley, R. S., D. Bucke, and R. H. Richards 1984. Proliferative kidney disease of salmonid fish: a review. *Journal of Fish Disease* **7**: 363−377.

Clifton-Hadley, R. S., R. H. Richards, and D. Bucke 1986. Proliferative Kidney disease (PKD) in rainbow trout *Salmo gairdneri*: Further observations on the effects of water temperature. *Aquaculture* 55:165-171.

Collins, C. M., R. Kerr, R. McIntosh, and M. Snow 2010. Development of a real-time PCR assay for the identification of *Gyrodactylus* parasites infecting salmonids in northern Europe. *Diseases of Aquatic Organisms* **90**:135-142.

Corbeil S., K. A. McCol, and M. J. S. Crane 2003. Development of a TaqMan quantitative PCR assay for the identification of *Piscirickettsia salmonis*. *Bulletin of the European Association of Fish Pathologists* **23**:95 -101.

Crozier, L. G., A. P. Hendry, P. W. Lawson, T. P. Quinn, N. J. Mantua, J. Battin, R. G. Shaw, *et* *al.* 2008. Potential responses to climate change in organisms with complex life histories: evolution and plasticity in Pacific salmon. *Evolutionary Applications* **1**:252-270.

de Kinkelin, P., M. Bearzotti-Le Berre, and J. Bernard 1980. Viral hemorrhagic septicemia of Rainbow trout: selection of a thermoresistant virus variant and comparison of polypeptide synthesis with the wildtype virus strain. *Journal of Virology* **36**:652-658.

Dobos, P., and T. E. Roberts 1983. The molecular biology of infectious pancreatic necrosis virus: a review. *Canadian Journal of Microbiology* **29**:377-384.

Dorson M., E. Quillet, M. G. Hollebecq, C. Torhy, and B. Chevassus 1995. Selection of rainbow trout resistant to viral haemorrhagic septicaemia virus and transmission of resistance by gynogenesis. *Veterinary Research* **26**:361–368.

Duchaud, E., M. Boussaha, V. Loux, J. F. Bernardet, C. Michel, B. Kerouault, S. Mondot et al. 2007. Complete genome sequence of the fish pathogen *Flavobacterium psychrophilum*. *Nature Biotechnology* **25**:763−769.

Duesund, H., S. Nylund, K. Watanabe, K. F. Ottem, and A. Nylund 2010. Characterization of a VHS virus genotype III isolated from rainbow trout (*Oncorhynchus mykiss*) at a marine site of the west coast of Norway. *Virology Journal* **7**:19.

Egidius, E., R. Wiik, K. Andersen, K. A. Hoff, and B. Hjeltnes 1986. *Vibrio salmonicida* sp. nov., a new fish pathogen. *International Journal of Systematic Bacteriology* **36**:518-520.

Elliott, D. G., L. J. Applegate, A. L. Murray, M. K. Purcell, and C. L. McKibben 2013. Bench-top validation testing of selected immunological and molecular *Renibacterium salmoninarum* diagnostic assays by comparison with quantitative bacteriological culture. *Journal of Fish Diseases* **36**:779–809.

El-Matbouli, M., T. S. McDowell, D. B Antonio, K. B Andree, and R. P Hedrick 1998. Effect of water temperature on the development, release and survival of the triactinomyxon stage of *Myxobolus cerebralis* in it’s oligachaete host. *International Journal of Parasitology* **29**:627-641.

Emmerich, R., and C. Weibel 1894. Über eine durch Bacterien erzeugte Seuche unter den Forellen. *Archiv fur Hygiene und Bakteriologie* **21**:1–21.

Erickson, J. D. 1965. Report on the problem of *Ichthyosporidium* in rainbow trout. *The Progressive Fish-Culturist* **27**:179-184.

Evelyn, T. P. T., and G. S. Traxler 1978. Viral erythrocytic necrosis: natural occurrence in Pacific salmon and experimental transmission. *Journal of the Fisheries Board of Canada* **35**:903-907.

Evelyn, T. P. T., M. L. Kent, and T. T. Poppe 1998. Bacterial diseases. In: M. L. Kent, and T. T. Poppe, eds. *Diseases of Seawater Netpen-reared Salmonid Fishes*, pp. 17-34. Pacific Biological Station, Fisheries and Oceans Canada, Nanaimo, British Columbia.

Ewart, K. V., J. C. Belanger, J. Williams, T. Karakach, S. Penny, S. C. M. Tsoi, R. C. Richards, and S. E. Douglas 2005. Identification of genes differentially expressed in Atlantic salmon (*Salmo salar*) in response to infection by *Aeromonas salmonicida* using cDNA microarray technology. *Developmental and Comparative Immunology* **29**:333–347.

Ewart, K. V., J. Williams, R. C. Richards, J. W. Gallant, K. Melville, and S. E. Douglas 2008. The early response of Atlantic salmon (*Salmo salar*) macrophages exposed in vitro to *Aeromonas salmonicida* cultured in broth and in fish. *Developmental and Comparative Immunology* **32**:380–390.

Falk, K., E. Namork, E. Rimstad, S. Mjaaland, and B. H. Dannevig 1997. Characterization of infectious salmon anemia virus, an orthomyxo-like virus isolated from Atlantic salmon (*Salmo salar* L.). *Journal of Virology* **71**:9016-9023.

Ferguson, H. W., T. Poppe, and D. J. Speare 1990. Cardiomyopathy in farmed Norwegian salmon. *Diseases of Aquatic Organisms* **8**:225–231.

Ferguson, J. A., J. Romer, J. C. Sifneos, L. Madsen, C. B. Schreck, M. Glynn, and M. L. Kent 2012. Impacts of multispecies parasitism on juvenile coho salmon (*Oncorhynchus* *kisutch*) in Oregon. *Aquaculture* **362-363**:184–192.

Finstad, O. W., K. Falk, M. Lovoll, O. Evensen, and R. Rimstad 2012. Immunohistochemical detection of piscine reovirus (PRV) in hearts of Atlantic salmon coincides with the course of heart and skeletal muscle inflammation (HSMI). *Veterinary Research* **43**:27.

Foltz, J. R., K. P. Plant, K. Overturf, K. Clemens and M. S. Powell 2009. Detection of *Nucleospora salmonis* in steelhead trout, *Oncorhynchus mykiss* (Walbaum), using quantitative polymerase chain reaction (qPCR). *Journal of Fish Diseases* **32**:551–555.

Foott, J. S., R. Stone, E. Wiseman, K. True, and K. Nichols 2007. Longevity of *Ceratomyxa* *shasta* and *Parvicapsula* *minibicornis*: Actinospore infectivity in the Klamath River. *Journal of Aquatic Animal Health* **19**:77-83.

Frans, I., C. W. Michiels, P. Bossier, K. A. Willems, B. Lievens, and H. Rediers 2011. *Vibrio* *anguillarum* as a fish pathogen: virulence factors, diagnosis and prevention. *Journal of Fish Diseases* **34**:643-661.

Funk V. A., M. Raap, K. Sojonky, S. Jones, J. Robinson, C. Falkenberg, and K.M. Miller 2007. Development and validation of an RNA- and DNA-based quantitative PCR assay for determination of *Kudoa thyrsites* infection levels in Atlantic salmon Salmo salar. *Diseases of Aquatic Organisms* **75**:239-249.

Gerrit, T. A. A. Krasnov, P. Nilsen, M. Alarcon, S. Afanasyev, M. Rode, H. Takle et al. 2011. Transcriptome profiling of immune responses to cardiomyopathy syndrome (CMS) in Atlantic salmon. *BMC Genomics* **12**:459.

Gjedrem, T. and H. M. Gjøen 1995. Genetic variation in susceptibility of Atlantic salmon, *Salmo salar* L., to furunculosis, BKD and cold water vibriosis. *Aquaculture Research* **26**:129-134.

Gjedrem, T., R. Salte, and H. M. Gjoen 1991. Genetic variation in susceptibility of Atlantic salmon to furunculosis. *Aquaculture* **97**:1-6.

Gjøen H.M., T. Refstie, O. Ulla, and B. Gjerde 1997. Genetic correlations between survival of Atlantic salmon in challenge and field tests. *Aquaculture* **158**:277–288.

Glenn, R. A., P. W. Taylor, and K. C. Hanson 2011. The use of a real-time PCR primer/probe set to observe infectivity of *Yersinia ruckeri* in Chinook salmon, *Oncorhynchus tshawytscha* (Walbaum), and steelhead trout, *Oncorhynchus mykiss* (Walbaum). *Journal of Fish Diseases* **34:**783–791.

Graham, D. A., E. Fringuelli, H. M. Rowley, D. Cockerill, D. I. Cox, T. Turnbull, H. Rodger, *et al.* 2012. Geographical distribution of salmonid alphavirus subtypes in marine farmed Atlantic salmon, *Salmo salar* L., in Scotland and Ireland. *Journal of Fish Diseases* **35**:755–765.

Hallett S. L., and J. L. Bartholomew 2006. Application of a real-time PCR assay to detect and quantify the myxozoan parasite Ceratomyxa shasta in river water samples. Diseases of Aquatic Organisms 71:109-118.

Hallett S. L., and J. L. Bartholomew 2009. Development and application of a duplex QPCR for river water samples to monitor the myxozoan parasite *Parvicapsula minibicornis*. *Diseases of Aquatic Organisms* **86**:39-50.

Hallett, S. L., R. A. Ray, C. N. Hurst, R. A. Holt, G. R. Buckles, S. D. Atkinson, and J. L. Bartholomew 2012. Density of the waterborne parasite *Ceratomyxa shasta* and its biological effects on salmon. *Applied and Environmental Microbiology* **78**:3724–3731.

Haney, D. C., D. A. Hursh, M. C. Mix, and J. R. Winton 1992. Physiological and hematological changes in chum salmon artificially infected with erythrocytic necrosis virus. *Journal of Aquatic Animal Health* 4:48-57.

Harun, N. O., T. Wang, and C. J. Secombes 2011. Gene expression proviling in naïve and vaccinated rainbow trout after *Yersinia ruckeri* infection: Insights into the mechanisms of protection seen in vaccinated fish. 2011. *Vaccine* **29**: 4388-4399.

Haugland, Ø., A.B. Mikalsen, P. Nilsen, K. Lindmo, B.J. Thu, T.M. Eliassen, N. Roos, et al. 2011. Cardiomyopathy syndrome of Atlantic salmon (*Salmo salar* L.) is caused by a double-stranded RNA virus of the Totiviridae family. *Journal of Virology* **85**:5275-5286.

Henryon, M., P. Berg, N. J. Olesen,T. E. Kjaer, W. J. Slierendrecht, A. Jokumsen, and I. Lund 2005. Selective breeding provides an approach to increase resistance of rainbow trout (*Oncorhynchus mykiss*) to the diseases, enteric redmouth disease, rainbow trout fry syndrome, and viral haemorrhagic septicaemia. *Aquaculture* **250**:621–636.

Herath, T. K., J. E. Bron, K. D. Thompson, J. B. Taggart, A. Adams, J. H. Ireland, and R. H. Richards 2012. Transcriptomic analysis of the host response to early stage salmonid alphavirus (SAV-1) infection in Atlantic salmon (*Salmo salar* L.). *Fish and Shellfish Immunology* **32**:796-807.

Hetrick, F. M., J. L. Fryer, and M. D. Knittel 1979. Effect of water temperature on the infection of rainbow trout *Salmo gairdneri* Richardson with infectious haematopoietic necrosis virus. *Journal of Fish Diseases* **2**:253-257.

Holt, R. A., J. E. Sanders, J. L. Zinn, J. L. Fryer, and K. S. Pilcher 1975. Relation of water temperature to *Flexibacter columnaris* infection in steelhead trout (Salmo gairdneri), coho (*Oncorhynchus kisutch*) and chinook (*O. tshawytscha*) salmon. *Journal of the Fisheries Board of Canada* **32**:1553-1559.

Houston, R. D., A. Gheyas, A. Hamilton, D. R. Guy, A. E. Tinch, J. B. Taggart, B. J. McAndrew, *et al.* 2008a. Detection and confirmation of a major QTL affecting resistance to infectious pancreatic necrosis (IPN) in Atlantic salmon (Salmo salar). *Developmental Biology* **132**:199–204

Houston, R. D., C. S. Haley, A. Hamilton, D. R. Guy, A. E. Tinch, J. B. Taggart, B. J. McAndrew *et al.* 2008b. Major quantitative trait loci affect resistance to infectious pancreatic necrosis in Atlantic Salmon (Salmo salar). *Genetics* **178**:1109-1115.

Houston, R.D., C. S. Haley, A. Hamilton , D. R. Guy, J. C. Mota-Velasco, A. A. Gheyas, A. E. Tinch, *et al.* 2010. The susceptibility of Atlantic salmon fry to freshwater infectious pancreatic necrosis is largely explained by a major QTL. *Heredity* **105**:318–327.

Jacobson, K. C., D. Teel, D. M. Van Doornik, and E. Casillas 2008. Parasite-associated mortality of juvenile Pacific salmon caused by the trematode *Nanophyetus salmincola* during early marine residence. *Marine Ecology Progress Series* **354**:235-244.

Johnsen, B. O., and A. J. Jensen 1991. The *Gyrodactylus* story in Norway. *Aquaculture* **98**:289–302.

Johansen, L-H., and A.-I. Sommer 2001. Infectious pancreatic necrosis virus infection in Atlantic salmon Salmo salar post-smolts affects the outcome of secondary infections with infectious salmon anaemia virus or *Vibrio salmonicida*. *Diseases of Aquatic Organisms* **47**:109–117.

Jones S., G. Prosperi-Porta, and S. Dawe 2006. A new parvicapsulid (Myxosporea) species in adult pink salmon, *Oncorhynchus gorbuscha*, from the Quinsam River, British Columbia, *Canada. Journal of Parasitology* **92**:1313–1318.

Jones, S. R., G. Prosperi‐Porta, and E. Kim 2012. The diversity of Microsporidia in parasitic copepods (Caligidae: Siphonostomatoida) in the Northeast Pacific ocean with description of *Facilispora margolisi*, n. sp. and a new family Facilisporidae n. fam. *Journal of Eukaryotic Microbiology* **59**:206-217.

Jonstrup, S. P., S. Kahns, H. F. Skall, T. S. Boutrup, and N. J. Olesen 2013. Development and validation of a novel Taqman-based real-time RT-PCR assay suitable for demonstrating freedom from viral haemorrhagic septicaemia virus. *Journal of Fish Diseases* **36**:9-23.

Jørgensen, S. M., S. Afanasyev, and A. Krasnov 2008. Gene expression analyses in Atlantic salmon challenged with infectious salmon anemia virus reveal differences between individuals with early, intermediate and late mortality. *BMC Genomics* **9**:179.

Jørgensen, A., A. Nylund, V. Nikolaisen, S. Alexandersen, and E. Karlsbakk 2011. Real‐time PCR detection of Parvicapsula pseudobranchicola (Myxozoa: Myxosporea) in wild salmonids in Norway. *Journal of Fish Diseases* **34**:365-371.

Karlsen, M., K. Hodneland, C. Endresen, and A. Nylund 2006. Genetic stability within the Norwegian subtype of salmonid alphavirus (family Togaviridae). *Archives of Virology* **151**:861–874.

Karlsen, M., B. Gjerset, B., T. Hansen, T., and A. Rambaut 2013. Multiple introductions of salmonid alphavirus from a wild reservoir have caused independent and self-sustainable epizootics in aquaculture. *Journal of General Virology*: published ahead of print September 23, 2013, doi:10.1099/vir.0.057455-0.

Keeling, S. E., C. L. Brosnahan, C. Johnston, R. Wallis, N. Gudkovs and W. L. McDonald 2013. Development and validation of a real-time PCR assay for the detection of *Aeromonas salmonicida*. *Journal of Fish Diseases* **36**:495-503.

Kelley, G.O., F.J. Zagmutt-Vergara, C.M. Leutenegger, M.A. Adkison, D.V. Rava, and R.P. Hedrick 2004. Identification of a serine protease gene expressed by *Myxobolus* *cerebralis* during development in rainbow trout *Oncorhynchus* *mykiss*. *Diseases of Aquatic Organisms* **59**:235-248.

Kent, M. 2011. Infectious diseases and potential impacts on survival of Fraser River sockeye salmon. Cohen Commission Technical Report 1: 58 pp. Vancouver, B.C. [www.cohencommission.ca](http://www.cohencommission.ca)

Kent, M. L., S. C. Dawe, and D. J. Speare 1995. Transmission of *Loma salmonae* (Microsporea) to Chinook salmon in sea water. *Canadian Veterinary Journal* **36**:98-101.

Kibenge, M. J., T. Iwamoto, Y. Wang, A. Morton, M.G. Godoy, and F.S. Kibenge 2013. Whole-genome analysis of piscine reovirus (PRV) shows PRV represents a new genus in family Reoviridae and its genome segment S1 sequences group it into two separate sub-genotypes. *Virology Journal* **10**:230.

Kjøglum, S., M. Henryon, T. Aasmundstad, and I. Korsgaard 2008. Selective breeding can increase resistance of Atlantic salmon to furunculosis, infectious salmon anaemia and infectious pancreatic necrosis. *Aquaculture Research* **39**:498-505.

Kocan, R., P. Hershberger, J. Winton 2004. Ichthyophoniasis: An emerging disease of Chinook salmon in the Yukon River. *Journal of Aquatic Animal Health* **16**:58-72.

Kocan, R., P. Hershberger, G. Sanders, and J. Winton 2009. Effects of temperature on disease progression and swimming stamina in *Ichthyophonus*‐infected rainbow trout, *Oncorhynchus mykiss* (Walbaum). *Journal of Fish Diseases* **32**:835-843.

Kongtorp, R.T., T. Taksdal, and A. Lyngøy 2004. Pathology of heart and skeletal muscle inflammation (HSMI) in farmed Atlantic salmon *Salmo salar*. *Diseases of Aquatic Organisms* **59**:217-224.

Kongtorp, R. T. 2008. Heart and skeletal muscle inflammation (HSMI) in Atlantic salmon, Salmo salar: pathology, pathogenesis and experimental infection. PhD thesis. Unipub AS Oslo, pp.1-87.

Korsnes, K., M. Devold, A. H. Nerland, and A. Nylund 2005. Viral encephalopathy and retinopathy (VER) in Atlantic salmon *Salmo salar* after intraperitoneal challenge with a nodavirus from Atlantic halibut *Hippoglossus hippoglossus*. *Diseases of Aquatic Organisms* **68**:7–15.

Krasnov, A., G. Timmerhaus, B. L. Schiotz, J. Torgersen, S. Afanasyev, D. Iliev, J. Jørgensen, *et al.* 2011. Genomic survey of early responses to viruses in Atlantic salmon, *Salmo salar* L. *Molecular Immunology* **49**:163-174.

Kvellestad, A., K. Falk, S. M. R. Nygaard, K. Flesjå, and J. A. Holm 2005. Atlantic salmon paramyxovirus (ASPV) infection contributes to proliferative gill inflammation (PGI) in seawater reared *Salmo salar*. *Diseases of Aquatic Organisms* **67**:47–54.

LaPatra, S. E., J. L. Fryer, W. H. Wingfield, and R. P. Hedrick 1989. Infectious hematopoietic necrosis virus (IHNV) in coho salmon. *Journal of Aquatic Animal Health* **1**:277–280.

Larenas, J. J., J. Bartholomew, O. Troncoso, S. Fernández, H. Ledezma, N. Sandoval, P. Vera *et al.* 2003. Experimental vertical transmission of *Piscirickettsia salmonis* and in vitro study of attachment and mode of entrance into the fish ovum. *Diseases of Aquatic Organisms* **56**:25–30.

Larsson, T., A. Krasnov, J. Lerfall, T. Taksdal, M. Pedersen, and T. Morkore 2012. Fillet quality and gene transcriptome profiling of heart tissue of Altantic salmon with pancreas disease (PD). *Aquaculture* **330**:82-91.

Lee, D.-Y., K. Shannon, and L. A. Beaudette 2006. Detection of bacterial pathogens in municipal wastewater using an oligonucleotide microarray and real-time quantitative PCR. *Journal of Microbiological Methods* **65**:453–467.

Leeds, T. D. , J. T. Silverstein, G. M. Weber, R. L. Vallejo, Y. Palti, C. E. Rexroad III, J. Evenhuis, *et al.* 2010. Response to selection for bacterial cold water disease resistance in rainbow trout. *Journal of Animal Science* **88**:1936-1946.

Lloyd, S. J., S. E. LaPatra, K. R. Snekvik, K. D. Cain, and D. R. Call 2011. Quantitative PCR demonstrates a positive correlation between a *Rickettsia*-like organism and severity of strawberry disease lesions in rainbow trout, *Oncorhynchus mykiss* (Walbaum). *Journal of Fish Diseases* **34**:701–709.

Løvoll, M., J. Wiik-Nielsen, S. Grove, C.R. Wiik-Nielsen, A. B. Kristoffersen, R. Faller, T. Poppe, *et al.* 2010. A novel totivirus and piscine reovirus (PRV) in Atlantic salmon (*Salmo salar*) with cardiomyopathy syndrome (CMS). *Virology Journal* **7**:309.

Magor, B.G. 1987. First report of *Loma* sp. (Microsporida) in juvenile coho salmon (Oncorhynchus kisutch) from Vancouver Island, British Columbia. *Canadian Journal of Zoology* **65**:751-752.

Malmberg, G. 1993. *Gyrodactylidae* and gyrodactylosis of Salmonidae. *Bulletin of the European Association of Fish Pathologists* **328**:5–46.

Marjara, I. S., N. Bain, and Ø. Evensen 2011. Naïve Atlantic salmon (*Salmo Salar* L.) surviving a lethal challenge with infectious pancreatic necrosis virus (IPNV) shows upregulation of antiviral genes in head-kidney, including Vig-2. *Aquaculture* **318**:300–308.

Markussen, T., M. K. Dahle, T. Tengs, M. Løvoll, Ø. W. Finstad, C. R. Wiik-Nielsen, S. Grove, *et* *al* 2013. Sequence analysis of the genome of piscine orthoreovirus (PRV) associated with heart and skeletal muscle inflammation (HSMI) in Atlantic salmon (*Salmo salar*). PLOS One 8: e70075

Markwardt, N. M., Y. M. Gocha, and G. W. Klontz 1989. A new application for Coomassie Brilliant Blue agar: detection of *Aeromonas salmonicida* in clinical samples. *Diseases of Aquatic Organisms* **6**:231-233.

McCullough, D. A. 1999. A Review and Synthesis of Effects of Alterations to the Water Temperature Regime on Freshwater Life Stages of Salmonids, with Special Reference to Chinook Salmon. U.S. Environmental Protection Agency, Region 10, Seattle, Washington.

McLoughlin M. F., H. M. Rowley, and C. E. Doherty 1998. A serological survey of salmon pancreas disease virus (SPDV) antibodies in farmed Atlantic salmon, *Salmo salar* L. *Journal of Fish Diseases* **21**:305–307.

McVicar, A. H. 1999. *Ichthyophonus* and related organisms. In: P. T. K. Woo, and D. W. Bruno, eds. *Fish Diseases and Disorders*, Volume 3. Viral, Bacterial and Fungal Infections. pp. 661–687. CAB International, Oxon, United Kingdom.

Mesa, M. G., A. G. Maule, T. P. Poe, and C .B. Schreck 1999. Influence of bacterial kidney disease on smoltification in salmonids: is it a case of double jeopardy? *Aquaculture* **174**:25-41.

Meyers, T. R. 2006. Standard necropsy procedures for finfish. National Wild Fish Health Survey–Laboratory Procedures Manual.

Miller, K., G. Traxler, K. Kaukinen, S. Li, J. Richard, and N. Ginther 2007. Salmonid host response to infectious hematopoietic necrosis (IHN) virus: cellular receptors, viral control, and novel pathways of defence. *Aquaculture* **272**:S217-S237.

Miyamoto, N., and M. Eguchi 1996. Response to low osmotic stress in a fish pathogen, *Vibrio* *anguillarum*. *Federation of European Microbiological Societies Microbiology Ecology* **22**:225-231.

Mo, T. A. 1994. Status of *Gyrodactylus* *salaris* problems and research in Norway. In: A. W. Pike and J. W. Lewis, eds., *Parasitic Diseases of Fish*. Samara Publishing Limited, Samara House, Tresaith, Dyfed, U.K. pp. 43-56.

Moen, T., K. T. Fjalestad, H. Munck, and L. Gomez-Raya 2004. A multi-stage testing strategy for detection of quantitative trait loci affecting disease resistance in Atlantic salmon. *Genetics* **167**:851–858.

Moen, T., A. K. Sonesson, B. Hayes, S. Lien, H. Munck, and T. H. E. Meuwissen 2007. Mapping of a quantitative trait locus for resistance against infectious salmon anemia in Atlantic salmon (*Salmo salar*): comparing survival analysis with analysis on affected/resistant data. *BMC Genetics* **8**:53.

Moles, A., and J. Heifetz 1998. Effects of the brain parasite *Myxobolus arcticus* on sockeye salmon. *Journal of Fish Biology* **52**:146-151.

Moran, J. D. W., D. J. Whitaker, and M. L. Kent 1999. A review of the myxosporean genus *Kudoa* Meglitsch, 1947, and its impact on the international aquaculture industry and commercial fisheries. *Aquaculture* **172**:163-196.

Morrison, R. N., G. A. Cooper, B. F. Koop, M. L. Rise, A. R. Bridle, M. B. Adams, and B. F. Nowak 2006. Transcriptome profiling the gills of amoebic gill disease (AGD)-affected Atlantic salmon (*Salmo salar* L.): a role for tumor suppressor p53 in AGD pathogenesis? *Physiological Genomics* **26**:15-34.

Norris, A., L. Foyle, and J. Ratcliff 2008. Heritability of mortality in response to a natural pancreas disease (SPDV) challenge in Atlantic salmon, *Salmo salar* L., post-smolts on a West of Ireland sea site. *Journal Fish Diseases* **31**:913–920.

Novoa, B., S. Mackenzie, and A. Figueras 2010. Inflammation and innate immune response against viral infections in marine fish. *Current Pharmaceutical Design* **16:**4175-4184.

Nylund, A., T. Hovland, K. Hodneland, F. Nilsen, and P. Lovik 1994. Mechanisms for transmission of infectious salmon anaemia (ISA). *Diseases of Aquatic Organisms* **19**:95-100.

Nylund, A., Plarre, H., Hodneland, K., Devold, M., Aspehaug, V., Aarseth, M., Koren, C., *et al.* 2003. Haemorrhagic smolt syndrome (HSS) in Norway: pathology and associated virus-like particles. *Diseases of Aquatic Organisms* **54**:15–27.

Nylund, A., E. Karlsbakk, P. A. Sæther, C. Koren, T. Larsen, B. D. Nielsen, A. E. Brøderud, et al. 2005. *Parvicapsula pseudobranchicola* (Myxosporea) in farmed Atlantic salmon *Salmo salar*: tissue distribution, diagnosis and phylogeny. *Diseases of Aquatic Organisms* **63**:197-204.

Nylund A., K. Watanabe, S. Nylund, M. Karlsen, P.A. Sæther, C.E. Arnesen, and E. Karlsbakk 2008. Morphogenesis of salmonid gill poxvirus associated with proliferative gill disease in farmed Atlantic salmon (*Salmo salar*) in Norway. *Archives of Virology* **153**:1299-1309.

Nylund S., A. Nylund, K. Watanabe, C.E. Arnesen, and E. Karlsbakk 2010. *Paranucleospora* *theridion* n. gen., n. sp. (Microsporidia, Enterocytozoonidae) with a life cycle in the salmon louse (*Lepeophtheirus salmonis*, Copepoda) and Atlantic salmon (*Salmo salar*). *Journal of Eukaryote Microbiology* **57**:95-114.

Nylund, S., L. Andersen, I. Sævareid, H. Plarre, K. Watanabe, C. E. Arnesen, E. Karlsbakk, and A. Nylund 2011. Diseases of farmed Atlantic salmon *Salmo salar* associated with infections by the microsporidian *Paranucleospora theridion*. *Diseases of Aquatic Organisms* **94**:41-57.

Oivind, E., B. Husevag, and J. Goksoyr 1989. Presence of the fish pathogen *Vibrio salmonicida* in fish farm sediments. *Applied and Environmental Microbiology* **55**:2815-2818.

Olesen, N. J., N. Lorenzen, and P. E. V. Jørgensen 1991. Detection of rainbow trout antibody to Egtved virus by enzyme-linked immunosorbent assay (ELISA), immunofluorescence (IF), and plaque neutralization tests (50% PNT). *Diseases of Aquatic Organisms* **10**:31-38.

Ozaki, A., T. Sakamoto, S. Khoo, K. Nakamura, M. R. Coimbra, T. Akutsu, and N. Okamoto 2001. Quantitative trait loci (QTLs) associated with resistance/susceptibility to infectious pancreatic necrosis virus (IPNV) in rainbow trout (*Oncorhynchus mykiss*). *Molecular Genetics and Genomics* **265**:23–31.

Pacha, R. E., and E. J. Ordal 1963. Epidemiology of columnaris disease in salmon. Bacteriological Proceedings **63**:3-4.

Palacios, G., M. Lovoll, T. Tengs, M. Hornig, S. Hutchison, J. Hui, R.T. Kongtorp, R.T., et al. 2010. Heart and skeletal muscle inflammation of farmed salmon is associated with infection with a novel reovirus. *PLoS One* **5**:e11487

Pirhonen, J., C. B. Schreck, and A. Gannam 2000. Appetite of chinook salmon (*Oncorhynchus* *tshawytscha*) naturally infected with bacterial kidney disease. *Aquaculture* **189**:1-10.

Plarre, H., M. Devold, M. Snow, and A. Nylund 2005. Prevalence of infectious salmon anaemia virus (ISAV) in wild salmonids in western Norway. *Diseases of Aquatic Organisms* **66**:71-79.

Plumb, J. A., and L. A. Hanson 2011. *Health Maintenance and Principal Microbial Diseases of Cultured Fishes*, third edition, Wiley-Blackwell, Hoboken, NJ, 506 pp.

Price, C. S., and C. B. Schreck 2003. Effects of bacterial kidney disease on saltwater preference of juvenile spring Chinook salmon, *Oncorhynchus tshawystcha*. *Aquaculture* **222**:331-341.

Purcell, M. K., I. S. Marjara, W. Batts, G. Kurath, and J. D. Hansen 2006. Transcriptome analysis of rainbow trout infected with high and low virulence strains of Infectious hematopoietic necrosis virus. *Fish and Shellfish Immunology* **30**:84-93.

Quinn, T. P., C. C. Wood, L. Margolis, B. E. Riddell, and K. D. Hyatt 1987. Homing in wild sockeye salmon (*Oncorhynchus nerka*) populations as inferred from differences in parasite prevalence and allozyme allele frequencies. *Canadian Journal of Fisheries and Aquatic Sciences* **44**:1963-1971.

Quinn, T. P., and M. T. Kinnison 1999. Size-selective and sex-selective predation by brown bears on sockeye salmon. *Oecologia* **121(2):**273-282.

Rahimian, H. 1998. Pathology and morphology of *Ichthyophonus hoferi* in naturally infected fishes off the Swedish west coast. *Diseases of Aquatic Organisms* **34**:109-123.

Rahimian, H., and J. Thulin 1996. Epizootiology of *Ichthyophonus* *hoferi* in herring populations off the Swedish west coast. *Disease of Aquatic Organisms* **27**:187–195.

Ray, R. A., P. A. Rossignol, and J. L. Bartholomew 2010. Mortality threshold for juvenile Chinook salmon *Oncorhynchus tshawytscha* in an epidemiological model of *Ceratomyxa* *shasta*. *Diseases of Aquatic Organisms* **93**:63–70.

Reith, M. E., R. K. Singh , B. Curtis , J. M. Boyd , A. Bouevitch , J. Kimball , J. Munholland , et al. 2008. The genome of *Aeromonas salmonicida* subsp. salmonicida A449: insights into the evolution of a fish pathogen. *BMC Genomics* **9**:427.

Rise, M. L., S. R. M. Jones, G. D. Brown, K. R. von Schalburg, W. S. Davidson, and B. F. Koop 2004. Microarray analyses identify molecular biomarkers of Atlantic salmon macrophage and hematopoietic kidney response to *Piscirickettsia salmonis* infection. *Physiological* *Genomics* **20**: 21–35.

Rønneseth, A., H. I. Wergeland, M. Devik, O. Evensen, and E. F. Pettersen 2007. Mortality after IPNV challenge of Atlantic salmon (*Salmo salar*) differs based on developmental stage of fish or challenge route. *Aquaculture* **271**:100-111.

Rønneseth, A., E. F. Pettersen, and H. I. Wergeland 2012. Flow cytometry assay for intracellular detection of Infectious Pancreatic Necrosis virus (IPNV) in Atlantic salmon (*Salmo salar* L.) leucocytes. *Fish and Shellfish Immunology* **33**:1292–1302.

Rucker, R. R., W. J. Whipple, J. R. Parvin, and C. A. Evan 1953. A contagious disease of sockeye salmon possibly of virus origin. *Fishery Bulletin of the Fish and Wildlife Service* **54**:35–46.

Seshadri, R., S. W. Joseph, A. K. Chopra, J. Sha, J. Shaw, J. Graf, D. Haft, *et al.* 2006. Genome sequence of *Aeromonas hydrophila* ATCC 7966T: jack of all trades. *Journal of Bacteriology* **188**:8272–8282.

Shaw, R. W., M. L. Kent, M. V. Brown, C. M. Whipps, and M. L. Damson 2000. Experimental and natural host specificity of *Loma salmonae* (Microsporidia). *Diseases of Aquatic Organisms* **40**:131-136.

Sindermann, C. J. 1958. An epizootic in Gulf of Saint Lawrence fishes. *Transactions of the North American Wildlife Conference* **23**:349-360.

Sindermann, C. J., and J. F. Chenoweth 1993. The fungal pathogen *Ichthyophonus hoferi* in sea herring *Clupea harengus*: a perspective from the western North Atlantic. *International Council for Exploration of the Sea* CM 1993/F:**41** (ref Pelagic Fish Committee), pp. 1-39.

Skall, H.F., N. J. Olesen and S. Mellergaard 2005. Viral haemorrhagic septicaemia virus in marine fish and its implications for fish farming – a review. *Journal of Fish Diseases* **28**:509–529.

Snow, M., P. McKay, and I. Matejusova 2009. Development of a widely applicable positive control strategy to support detection of infectious salmon anaemia virus (ISAV) using Taqman real-time PCR. *Journal of Fish Diseases* **32**:151-156.

Soleng, A., T. A. Bakke, and L. P. Hansen 1998. Potential for dispersal of *Gyrodactylus salaris* (Platyhelminthes, Monogenea) by sea-running stages of the Atlantic salmon (*Salmo* *salar*): field and laboratory studies. *Canadian Journal of Fisheries and Aquatic Sciences* **55**:507–514.

Stephen, C., T. Stitt, J. Dawson-Coates and A. McCarthy 2011. Assessment of the potential effects of diseases present in salmonid enhancement facilities on Fraser River sockeye salmon. Cohen Commission Technical Report 1A: 180p. Vancouver, B.C. available at: www.cohencommission.ca

St-Hilaire, S., C. Ribble, G. Traxler, T. Davies, and M. L. Kent 2001. Evidence for a carrier state of infectious hematopoietic necrosis virus in chinook salmon *Oncorhynchus* *tshawytscha*. *Diseases of Aquatic Organisms* **46**:173–179.

Stocking, R. W., R. A. Holt, J. S. Foott, J. L. Bartholomew 2006. Spatial and temporal occurrence of the salmonid parasite *Ceratomyxa shasta* in the Oregon-California Klamath River Basin. *Journal of Aquatic Animal Health* **18**:194-202.

Storset, A., C. M. Strand, C., M. Wetten, S. Kjøglum, and A. Ramstad 2007. Response to selection for resistance against infectious pancreatic necrosis in Atlantic salmon (*Salmo* *salar* L.). *Aquaculture* **272**:S62-S68.

Suzuki, K., and D. K. Sakai 2007. Real-time PCR for quantification of viable *Renibacterium salmoninarum* in chum salmon *Oncorhynchus keta*. *Diseases of Aquatic Organisms* **74**:209-223.

Thorud, K. and H. O. Djupvik 1988. Infectious anaemia in Atlantic Salmon (*Salmo salar* L.) . *Bulletin of the European Association of Fish Pathology* **8**:109-111.

Tierney, K.B., and A.P. Farrell 2004. The relationships between fish health, metabolic rate, swimming performance and recovery in return-run sockeye salmon, *Oncorhynchus* *nerka* (Walbaum). *Journal of Fish Diseases* **27**:663-671.

Timmerhaus, G., A. Krasnov, P. Nilsen, M. Alarcon, S. Afanasyev, M. Rode, H. Takle, et al. 2011. Transcriptome profiling of immune responses of cardiomyopathy syndrome (CMS)

in Atlantic salmon. BMC Genomics 12:459.

Traxler, G. S., J. R. Roome, K. A. Lauda and S. LaPatra 1997. Appearance of infectious hematopoietic necrosis virus (IHNV) and neutralizing antibodies in sockeye salmon *Oncorhynchus nerka* during their migration and maturation period. *Diseases of Aquatic Organisms* **28**:31-38.

Traxler, G. S., M. L. Kent, and T. T. Poppe 1998. Viral diseases. In: M. L. Kent and T. T. Poppe, eds. Diseases of seawater netpen-reared salmonid fishes. pp. 36-45. Fisheries and Oceans, Science Branch, Nanaimo, B.C.

Uno, M. 1990. Effects of seawater acclimation on juvenile salmonids infected with *Tetraonchus* (Monogenea) and *Ichthyophonus* (Phycomycetes). *Fish Pathology* **25**:15-19.

Verrier, E. R., C. Langevin, A. Benmansour, and P. Boudinot 2011. Early antiviral response and virus-induced genes in fish. *Developmental and Comparative Immunology* **35**: 1204–1214.

Verrier, E. R., M. Dorson, S. Mauger, C. Torhy, C. Ciobotaru, C. Hervet, N. Dechamp, et al. 2013a. Resistance to a rhabdovirus (VHSV) in Rainbow Trout: Identification of a major QTL related to innate mechanisms. *PloS One* **8** :e55302.

Verrier, E.R., A. Ehanno, S. Biacchesi, S. Le Guillou, N. Dechamp, P. Boudinot, M. Bremont, et al. 2013b. Lack of correlation between the genetic resistances to two rhabdovirus infections in rainbow trout. *Fish and Shellfish Immunology* **35**:9-17.

Wertheimer, A.C. and J.R. Winton 1982. Differences in susceptibility among three stocks of chinook salmon, *Oncorhynchus tshawytscha*, to two isolates of infectious hematopoietic necrosis virus. NOAA Technical Memorandum NMFS F/NWC 22. Washington, D.C.

Wiens, G. D., D. D. Rockey, Z. Wu, J. Chang, R. Levy, S. Crane, D. S. Chen, et al. 2008. Genome sequence of the fish pathogen *Renibacterium salmoninarum* suggests reductive evolution away from an environmental Arthrobacter ancestor. *Journal of Bacteriology* **190**:6970–6982.

Wiik-Nielsen, C. R., M. Løvoll, N. Sandlund, R. Faller, J. Wiik-Nielsen, and B. B. Jensen 2011. First detection of Piscine reovirus (PRV) in marine fish species. *Diseases of Aquatic Organi*sms **97**: 255-258.

Wiik-Nielsen, C. R., P. M. R. Ski, A. Aunsmo, and M. Løvoll 2012. Prevalence of viral RNA from piscine reovirus and piscine myocarditis virus in Atlantic salmon, *Salmo salar* L., broodfish and progeny. *Journal of Fish Diseases* **35**:169−171.

Winton, J. R., W. N. Batts, R. Deering, R. Brunson, K. Hopper, T. Nishizawa, and C. Stehr 1991. Characteristics of the first North American isolates of Viral hemorrhagic septicemia virus. pp. 43-50. In: Proceedings of the Second International Symposium on Viruses of Lower Vertebrates, July 29-31, Corvallis, Oregon.

Withler, R. E., and T. P. T. Evelyn 1990. Genetic variation in resistance to bacterial kidney disease within and between two strains of Coho salmon from British Columbia. *Transactions of the American Fisheries Society* **119**:1003–1009.

Wolf, K. 1988. Viral hemorrhagic septicemia. In: K. Wolf, ed. *Fish Viruses and Fish Viral Diseases*, pp. 217-249. Comstock Publishing Associates. Cornell University Press. Ithaca, NY.

Wood, E. M. and W. T. Yasutake 1956. Histopathology of kidney disease in fish. *American Journal of Pathology* 32:845–857.

Workenhe S. T., T. S. Hori, M. L. Rise, M. J. T. Kibenge, and F. S. B. Kibenge 2009. Infectious salmon anaemia virus (ISAV) isolates induce distinct gene expression responses in the Atlantic salmon (*Salmo salar*) macrophage/dendritic-like cell line TO, assessed using genomic techniques. *Molecular Immunology* **46**:2955-74.
